# Supplementary material for: Recruiting and retaining bachelor qualified nurses in German hospitals (BSN4Hospital): protocol of a mixed-methods design
Source: BMJ Open. 2023 Aug 10;13(8):e073879. doi: 10.1136/bmjopen-2023-073879 (PMC10423778; doi:10.1136/bmjopen-2023-073879)
Supplement: Supplementary data [file bmjopen-2023-073879supp001.pdf]

Technische Universität Berlin

Supplement 1. Interview guides: BSN4Hospital Study

Interview guide 1 (hospital managers, e.g. CNOs) in German

Für CNOs/Pflegedirektion in deutschen Krankenhäusern

Einstieg: *Der Anteil der Pflegepersonen mit mindestens Bachelor-Abschluss (auch hochschulisch qualifizierte Pflegefachpersonen genannt), ist in deutschen Krankenhäusern noch gering, im Vergleich zu anderen europäischen Ländern. Ich möchte darum gerne mehr über die Einbindung hochschulisch ausgebildeter Pflegender in Ihrem Krankenhaus erfahren. Vorab möchte ich noch einmal darauf hinweisen, dass ich mit „hochschulisch bzw. akademisch ausgebildet“, Pflegepersonen meine, die <u>mindestens</u> einen Bachelor-Abschluss haben, oder auch einen höheren Abschluss, also einen Masterabschluss, wie z.B. Advanced Practice Nurses (APN).*

1. Gibt es in Ihrem Haus eine Strategie oder einen Aktionsplan, um den Anteil des hochschulisch ausgebildete (akademisierten) Pflegefachpersonals zu erhöhen? Wenn ja, was sind die Inhalte?
   a) Wurden für Ihr Haus Ziele formuliert den Anteil hochschulisch ausgebildeter Pflegefachpersonen zu erhöhen? Wenn ja, kennen Sie dieses Ziel/ den Zielwert? (Prozent/ Bis wann?)
   b) Wie setzen Sie dieses Ziel um?
   c) Wie wird der Erfolg gemessen/ Wird ein Monitoring durchgeführt?
   d) Wissen Sie, wie viele akademisch ausgebildete Pflegefachpersonen in Ihrem Haus arbeiten? Hat sich der Anteil in den letzten Jahren verändert?

2. Gibt es in Ihrem Haus Maßnahmen <u>zur Rekrutierung und Einbindung von</u> akademisierten Pflegefachpersonen?
   a) Wie wurden die Maßnahmen ausgewählt und umgesetzt?
   b) Was sehen Sie als förderlich für die Rekrutierung/Gewinnung?
   c) Welche Hindernisse gibt es Ihrer Meinung nach?
   d) Was sollte verbessert werden?

3. Gibt es in Ihrem Haus Maßnahmen zur <u>längerfristigen Bindung</u> von akademisierten Pflegefachpersonen?
   a) Wie wurden die Maßnahmen ausgewählt und umgesetzt?
   b) Wie gut gelingt es aus Ihrer Sicht Ihrer Klinik hochschulisch ausgebildete Pflegefachpersonen längerfristig zu halten?
   c) Was sehen Sie als förderlich für die langfristige Bindung?
   d) Welche Hindernisse gibt es Ihrer Meinung nach?
   e) Was sollte verbessert werden?

4. Wie werden hochschulisch qualifizierte Pflegefachpersonen in Ihrem Haus in der Praxis eingesetzt?
   a) Gibt es spezielle Tätigkeitsbereiche für diese Pflegefachpersonen? Wenn ja, wie sehen diese aus/können Sie mir Beispiele nennen?

b) Gibt es Aufgaben, die ausschließlich von akademisierten Pflegefachpersonen in Ihrem Haus übernommen werden? Welche sind das?
c) Gibt es ein Mentoring-Programm/spezifisches Einarbeitungsprogramm für akademisiertes Pflegepersonal?

5. Was sind Ihrer Meinung nach die Hauptfaktoren/-bedingungen, warum sich akademisierte Pflegefachpersonen für die Arbeit in Ihrem Krankenhaus entscheiden?
a) Was bietet das Krankenhaus dieser Berufsgruppe?
b) Gibt es Angebote in Ihrem Haus, die besonders anziehend auf hochschulisch ausgebildete Pflegende wirken?
c) Was müsste noch verbessert werden?

Wir haben uns bisher über Ihr Haus unterhalten. Jetzt erweitern wir den Blick auf die Situation in Deutschland.

6. Was denken Sie, wie steht Ihr Haus im Vergleich zu anderen Krankenhäusern in Deutschland da (in Bezug auf die Rekrutierung und langfristige Bindung hochschulisch ausgebildeter Pflegefachpersonen)?
   a. Was läuft gut?
   b. Was läuft weniger gut?
   c. Was könnte insgesamt an deutschen Krankenhäusern verbessert werden, um hochschulisch ausgebildete Pflegefachpersonen zu rekrutieren und zu halten?
   d. Gibt es hinderliche Faktoren oder Rahmenbedingungen in Deutschland, die es den Krankenhäusern erschweren, akademisches Pflegefachpersonal zu gewinnen und zu halten?
   e. Gibt es förderliche Faktoren in Deutschland, die es den Krankenhäusern erleichtern, akademisches Pflegefachpersonal zu gewinnen und zu halten?

7. Was können deutsche Krankenhäuser aus den bisherigen Erfahrungen mit der Rekrutierung und Binding von akademisiertem Pflegepersonal für die Zukunft lernen?
   a. Was sollten deutsche Krankenhäuser generell bedenken, wenn Sie akademisches Pflegepersonal binden möchten?

8. Gibt es zum Schluss irgendetwas, was Sie noch hinzufügen möchten oder was Sie denken, das wichtig ist, für das Thema Gewinnung und Bindung hochschulisch ausgebildeter Pflegepersonen?

Ende des Interviews: *Ich danke Ihnen für Ihre Teilnahme. Wenn Sie nach diesem Interview noch Fragen haben sollten, oder Ihnen noch etwas einfällt, können Sie sich gerne an mich wenden - die Kontaktinformationen finden Sie auf dem Informationsblatt zur Teilnahme.*

Nach dem Interview: **Kurzfragebogen ausfüllen lassen**

Technische Universität Berlin

Interview guide 2 (in German) for BSN/MSN

Für Pflegepersonen mit BSN/ MSN in deutschen Krankenhäusern

Einstieg: *Der Anteil der Pflegepersonen mit mindestens Bachelor-Abschluss (auch hochschulisch qualifizierte Pflegefachpersonen genannt), ist in deutschen Krankenhäusern noch gering, im Vergleich zu anderen europäischen Ländern. Mich interessieren darum Ihre Erfahrungen und Motivation als akademisch ausgebildete*r Pflegende*r. Vorab möchte ich noch einmal darauf hinweisen, dass ich mit „hochschulisch bzw. akademisch ausgebildet", Pflegepersonen meine, die mindestens einen Bachelor-Abschluss haben, oder auch einen höheren Abschluss, also einen Masterabschluss wie z.B. Advanced Practice Nurses (APN).*

1. Als erstes würde mich interessieren, wie lange Sie schon in Ihrem Krankenhaus tätig sind?
   a. Welchen Abschluss haben Sie und seit wann?
   b. Warum haben Sie sich für einen Studienabschluss in der Pflege entschieden?
   c. Haben Sie vor weiter zu studieren/promovieren/habilitieren? Warum?

2. Warum arbeiten Sie in genau diesem Krankenhaus?
   a. Warum haben Sie sich für die Arbeit in diesem Krankenhaus entschieden?
      i. Gab es ausschlaggebende Faktoren, die Sie überzeugt haben?
      ii. Hat Sie etwas besonders angesprochen? Wenn ja, was?
   b. Können Sie mir sagen, was Sie besonders motiviert in Ihrem Krankenhause zu arbeiten und warum?
   c. Gibt es etwas, das Sie frustriert? Wenn ja, was ist das und warum?
   d. Wie lange beabsichtigen Sie in diesem Krankenhaus tätig zu sein?
      iii. Was Sind Ihre beruflichen Pläne?
      iv. Inwieweit wurde Ihre berufliche Zukunft mit der Leitungsebene/Pflegedirektion besprochen?

3. Haben Sie als Pflegeperson mit Bachelor/Master-Abschluss eine spezielle Rolle im Krankenhaus?
   a. Wie unterscheiden sich Ihre Tätigkeiten zu Pflegefachpersonen ohne Studium?
   b. Wie sieht Ihre Zusammenarbeit im Team aus?
   c. Wie zufrieden bzw. unzufrieden sind Sie mit Ihrer Rolle und warum?
   d. Was würden Sie gerne ändern?

4. Welche Maßnahmen sollte es in Ihrer Klinik geben, um mehr Pflegefachpersonen mit Bachelor- oder Masterabschluss zu gewinnen und längerfristig zu halten?
   a. Sollten sich die Maßnahmen zu Gewinnung von akademisiertem Pflegepersonal von den Maßnahmen zur langfristigen Bindung unterscheiden? Können Sie mir Beispiele nennen?
      i. Welche Themen sind wichtig, wenn es um die langfristige Bindung von akademischen Pflegefachpersonen geht?

Wir haben uns bisher über Ihre Erfahrungen in diesem Haus unterhalten. Jetzt erweitern wir den Blick auf die Situation in Deutschland.

5. Was denken Sie, wie steht Ihr Haus im Vergleich zu anderen Krankenhäusern in Deutschland da?

a. Haben Sie bereits in anderen Krankenhäusern gearbeitet?
b. Was war da anders?
c. Was lief gut/schlecht?

6. Was sollte sich in Deutschland ändern, um mehr hochschulisch ausgebildete Pflegefachpersonen für die klinische Pflege zu gewinnen?
   a. Welche Faktoren sind hinderlich/ förderlich?
   b. Was sollte verbessert werden und wie?

7. Abschließend würde ich Sie gerne bitten, sich vorzustellen, dass Sie auf der Suche nach einer neuen Stelle in einem anderen Krankenhaus sind. Was würden Sie sich in Bezug auf die neue Stelle und Tätigkeit wünschen?
   a. Was würde Sie überzeugen in einem Krankenhaus anzufangen?

8. Gibt es zum Schluss irgendetwas, das Sie noch hinzufügen möchten oder was Sie denken, das wichtig ist zum Thema Gewinnung und Bindung hochschulisch ausgebildeter Pflegefachpersonen?

Ende des Interviews: *Ich danke Ihnen für Ihre Teilnahme. Wenn Sie nach diesem Interview noch Fragen haben sollten, oder Ihnen noch etwas einfällt, können Sie sich gerne an mich wenden - die Kontaktinformationen finden Sie auf dem Informationsblatt zur Teilnahme.*

Nach dem Interview: **Kurzfragebogen ausfüllen lassen**

Technische Universität Berlin

Interview guide 3 (in German) for students

Für Studierende in einem Pflegestudiengang mit (erster) praktischer Erfahrung

Einstieg: *Der Anteil der Pflegepersonen mit mindestens Bachelor-Abschluss ist in deutschen Krankenhäusern noch gering, im Vergleich zu anderen europäischen Ländern. Mich interessieren darum Ihre Erfahrungen und Beweggründe,* **warum Sie sich für ein Pflegestudium** *entschieden haben. Vorab möchte ich noch einmal darauf hinweisen, dass ich mit „hochschulisch bzw. akademisch ausgebildet", Pflegepersonen meine, die mindestens einen Bachelor-Abschluss haben, oder auch einen höheren Abschluss, also einen Masterabschluss wie z.B. Advanced Practice Nurses (APN).*

1. Als erstes würde mich interessieren, wie lange Sie schon studieren?
    a. Welchen Abschluss streben Sie an und wann werden Sie diesen voraussichtlich erreichen?
    b. Warum haben Sie sich für einen Studiengang in der Pflege entschieden?
    c. Haben Sie bereits einen Berufs- oder Studienabschluss?
    d. Arbeiten Sie noch nebenbei oder haben Sie während Ihres Studiums nebenbei gearbeitet? Welchen Nebenjob üben Sie derzeit aus?

2. Hatten Sie schon Praxiseinsätze (wie viele Einsätze, unterschiedliche Organisationen, welche Fachbereiche?) Wie erleben Sie die Arbeit/Tätigkeit als Studierende im Krankenhaus?
    a. Was war/ist gut? Was nicht so gut?
    b. Auf welcher Station haben Sie zuletzt gearbeitet, bzw. wo sind Sie derzeit tätig? *(Fachrichtung/internistisch/chirurgisch/ITS/etc.)*?

3. Warum haben Sie sich für dieses Krankenhaus als Arbeitgeber (Praxispartner) entschieden?
    a. Gab es ausschlaggebende Faktoren, die Sie überzeugt haben?
    b. Hat Sie etwas besonders angesprochen? Wenn ja, was?
    c. Können Sie mir sagen, was Sie besonders motiviert, in Ihrem Krankenhause zu arbeiten und warum?
    d. Gibt es etwas, das Sie frustriert? Wenn ja, was ist das und warum?

4. In welchem Bereich möchten Sie nach Abschluss Ihres Studiums arbeiten?
    a. Wenn nicht klinischer Bereich, dann fragen, ob es für Sie infrage käme, in die klinische Pflege zu bleiben?
        i. Welche Bedingungen müssten dafür erfüllt sein?
        ii. Was müsste sich in deutschen Krankenhäusern ändern?
        iii. Was müsste sich generell in der Pflege ändern?
        iv. Was müsste sich in Deutschland ändern?
    b. Haben Sie vor weiter zu studieren/promovieren/habilitieren? Warum?

5. Haben Pflegefachpersonen mit Bachelor/Master-Abschluss eine spezielle Rolle in Ihrem Krankenhaus?
    a. Wie unterscheiden sich die Tätigkeiten zu Pflegefachpersonen ohne Studium?
    b. Welche Strategien, Maßnahmen oder Konzepte gibt es in Ihrem Krankenhaus, um Pflegefachpersonen mit Bachelor- oder Masterabschluss in der Pflegepraxis zu integrieren?
    c.

6. Welche Maßnahmen sollte es in Ihrer Klinik geben, um mehr Pflegefachpersonen mit Bachelor- oder Masterabschluss zu gewinnen und längerfristig zu halten?
    a. Sollten sich die Maßnahmen zu Gewinnung von hochschulisch ausgebildetem Pflegepersonal von den Maßnahmen zur langfristigen Bindung unterscheiden? Können Sie mir Beispiele nennen?
        I. Welche Themen sind wichtig, wenn es um die langfristige Bindung von akademischen Pflegefachpersonen geht? Probing: wie kann man diese Pflegefachpersonen länger an einer Klinik halten, d.h. mind. länger als 5 Jahre - oder sogar 10 Jahre?

Wir haben uns bisher über Ihre Erfahrungen in diesem Haus unterhalten. Jetzt erweitern wir den Blick auf die Situation in Deutschland.

7. Was denken Sie, wie steht Ihr Haus im Vergleich zu anderen Krankenhäusern in Deutschland da?
    d. Haben Sie bereits in anderen Krankenhäusern gearbeitet?
    e. Was war da anders?
    f. Was lief gut/schlecht?

8. Was sollte sich in Deutschland ändern, um mehr hochschulisch ausgebildete Pflegefachpersonen für die klinische Pflege zu gewinnen?
    c. Welche Faktoren sind hinderlich/ förderlich?
    d. Was sollte verbessert werden und wie?

9. Abschließend würde ich Sie gerne bitten, sich vorzustellen, dass Sie auf der Suche nach einer neuen Stelle in einem anderen Krankenhaus sind. Was würden Sie sich in Bezug auf die neue Stelle und Tätigkeit wünschen?
    b. Was würde Sie überzeugen in einem Krankenhaus anzufangen?

10. Gibt es zum Schluss irgendetwas, dass Sie noch hinzufügen möchten oder was Sie denken, das wichtig ist zum Thema Gewinnung und Bindung hochschulisch ausgebildeter Pflegefachpersonen?

Ende des Interviews: *Ich danke Ihnen für Ihre Teilnahme. Wenn Sie nach diesem Interview noch Fragen haben sollten, oder Ihnen noch etwas einfällt, können Sie sich gerne an mich wenden - die Kontaktinformationen finden Sie auf dem Informationsblatt zur Teilnahme.*

Nach dem Interview: **Kurzfragebogen ausfüllen lassen**

Technische Universität Berlin

Interview guide 4 (in German) for “leavers”

Pflegefachpersonen, die sich trotz Pflegestudium gegen eine klinische Tätigkeit entschieden haben oder sich gegen ein Pflegestudium (und gegen eine klinische Tätigkeit) entschieden haben.

Einstieg: *Der Anteil der Pflegepersonen, mit mindestens Bachelor-Abschluss, ist in deutschen Krankenhäusern noch gering, im Vergleich zu anderen europäischen Ländern. Mich interessieren darum Ihre Erfahrungen und Beweggründe, [JE nach Interviewteilnehmer*in anpassen] warum Sie für ein Pflegestudium aber gegen die klinische Tätigkeit/ warum Sie sich für ein Studium ohne Pflegebezug und gegen die klinische Tätigkeit entschieden haben. Vorab möchte ich noch einmal darauf hinweisen, dass ich mit „hochschulisch bzw. akademisch ausgebildet“, Pflegepersonen meine, die mindesten einen Bachelor-Abschluss haben, oder auch einen höheren Abschluss, also einen Masterabschluss wie z.B. Advanced Practice Nurses (APN).*

1. Sie haben eine Ausbildung in der Pflege und haben in der Vergangenheit als Pflegefachperson in der klinischen Pflege im Krankenhaus gearbeitet. Wie haben Sie die Arbeit/Tätigkeit als Pflegefachperson im Krankenhaus erlebt?
   a. Was war gut? Was nicht so gut?
   b. Warum haben Sie sich damals für diesen Beruf entschieden?
   c. Auf welcher Station haben Sie zuletzt gearbeitet *(Fachrichtung/internistisch/chirurgisch/ITS/etc.)*?
   d. Welche Strategien, Maßnahmen oder Konzepte gab es in den Krankenhäusern, in denen Sie zuvor gearbeitet haben, um Pflegefachpersonen mit Bachelor- oder Masterabschluss in der Pflegepraxis zu integrieren?

2. Sie haben ja auch einen Studienabschluss bzw. studieren derzeit, oder?
   a. Können Sie erläutern, was Ihre Beweggründe waren, ein Studium anzufangen?
   b. Was haben Sie studiert bzw. studieren Sie derzeit?
   c. Warum haben Sie sich für den Studiengang entschieden?

3. Die Anzahl der Studiengänge der Fachrichtung Pflege hat über die letzten Jahre hinweg zugenommen, auch die Anzahl der Absolvent*innen. Sie haben sich *[JE nach Interviewteilnehmer*in anpassen: für ein Pflegestudium bzw. für ein Studium ohne Pflegeschwerpunkt]* entschieden. Mich interessiert, was die Gründe dafür waren.
   a. Warum haben Sie sich für bzw. gegen einen Pflegestudiengang entschieden?
   b. Haben Sie vor weiter in dem von Ihnen gewählten Bereich zu studieren/promovieren/habilitieren? Warum?
   c. Falls Pflege und noch studierend: In welchem Bereich möchten Sie nach Abschluss Ihres Studiums arbeiten?
   d. Welchen Beruf üben Sie derzeit aus?

4. In Deutschland hat der Wissenschaftsrat bereits 2012 empfohlen, dass 10-20% eines Ausbildungsjahrgangs in der Pflege hochschulisch ausgebildet werden sollen. Wie ist Ihre Meinung dazu?

Maier CB, *et al. BMJ Open* 2023; 13:e073879. doi: 10.1136/bmjopen-2023-073879

a. Sollte Ihrer Meinung nach der Anteil hochschulisch ausgebildeter Pflegefachpersonen in der klinischen Pflege in Krankenhäusern erhöht werden? Wenn ja, warum?
b. Was sind Barrieren? Hinderliche Rahmenbedingungen?
c. Was sind förderliche Faktoren?
d. Was sollte sich in Deutschland/deutschen Krankenhäusern verändern? (Arbeitsumfeld; tarifliche Eingruppierung; Arbeitszeiten; Mitspracherecht; etc.)?

5. Welche Maßnahmen sollte es geben, um mehr Pflegefachpersonen mit Bachelor- oder Masterabschluss zu gewinnen und längerfristig zu halten?
   a. Was sollten Krankenhäuser tun, um akademisch ausgebildete Pflegefachpersonen erfolgreich zu rekrutieren?
   b. Welche Faktoren sind hinderlich/ förderlich?
   c. Was sollte sich in Deutschland verändern?
   d. Was sollte verbessert werden und wie?

6. Falls nicht in einem Pflegestudium: Abschließend möchte ich Sie fragen, ob es für Sie infrage käme, in die klinische Pflege zurückzukehren?
   a. Welche Bedingungen müssten dafür erfüllt sein?
   b. Was müsste sich in deutschen Krankenhäusern ändern?
   c. Was müsste sich generell in der Pflege ändern?
   d. Was müsste sich in Deutschland ändern?

7. Gibt es zum Schluss irgendetwas, das Sie noch hinzufügen möchten oder was Sie denken, das wichtig ist zum Thema Gewinnung und Bindung hochschulisch ausgebildeter Pflegefachpersonen?

Ende des Interviews: *Ich danke Ihnen für Ihre Teilnahme. Wenn Sie nach diesem Interview noch Fragen haben sollten, oder Ihnen noch etwas einfällt, können Sie sich gerne an mich wenden - die Kontaktinformationen finden Sie auf dem Informationsblatt zur Teilnahme.*

Nach dem Interview: **Kurzfragebogen ausfüllen lassen**

Technische Universität Berlin

Interview guide 5 (in German) for stakeholders/experts

Für Expert*innen und Stakeholders in Deutschland (z.B. Interessenvertretung, Patient*innenvertreter)

Einstieg: *Der Anteil hochschulisch ausgebildeter Pflegefachpersonen, also mit mindestens Bachelor-Abschluss, ist in deutschen Krankenhäusern noch gering, v.a. im Vergleich zu anderen europäischen Ländern. Es ist in Deutschland immer noch eine relativ neue Entwicklung: die Pflegestudiengänge, Einbindung in die klinische Praxis, Tätigkeitsfelder der Pflegefachpersonen mit Bachelor- und Masterabschluss, Vergütung, usw. Dies sind alles Themen, die derzeit aktuell sind. Ich möchte darum heute gerne mehr von Ihnen erfahren, welche Voraussetzungen geschaffen werden sollten, damit hochschulisch ausgebildete Pflegefachpersonen gerne und motiviert in der klinischen Pflege arbeiten möchten, wie Arbeitgeber (bspw. Krankenhäuser) attraktiv werden und diese Pflegefachpersonen auch längerfristig halten. Vorab möchte ich noch einmal darauf hinweisen, dass ich mit „hochschulisch bzw. akademisch ausgebildet", Pflegepersonen meine, die mindestens einen Bachelor-Abschluss haben, oder einen höheren Abschluss, also einen Masterabschluss wie z.B. Advanced Practice Nurses (APN).*

1. In Deutschland hat der Wissenschaftsrat bereits 2012 empfohlen, dass 10-20% eines Ausbildungsjahrgangs in der Pflege hochschulisch ausgebildet werden sollen. Dies ist ja nun bereits 10 Jahre her. Was hat sich Ihrer Meinung nach seither getan?
   a. Was lief gut?
   b. Was waren die Herausforderungen? Was lief nicht gut?
   c. Wie könnte verbessert werden?

2. Die Anzahl der Studiengänge Pflege hat ja über die letzten Jahre hinweg zugenommen, auch die Anzahl der Absolvent*innen. Jedoch arbeitet trotz allem nur ein geringer Prozentsatz in der klinischen Pflege (ca. 2-3% aller Pflegefachpersonen in Universitätsklinika). Sollte der Anteil erhöht werden? Wenn ja, warum und wie?
   a. Was sind Barrieren? Hinderliche Rahmenbedingungen?
   b. Gab es förderliche Faktoren?
   c. Was sollte in Deutschland verändert werden? (politische Rahmenbedingungen, gesetzlich, Studium, etc.)?

3. Wir möchten nun den Schwerpunkt auf Krankenhäuser legen: was sollten Krankenhäuser tun, um akademisch ausgebildete Pflegefachpersonen erfolgreich zu rekrutieren?
   a. Kennen Sie Beispiele guter Praxis?
   b. Was sollte in Deutschland an den Rahmenbedingungen verändert werden, um Krankenhäuser zu unterstützen? (politische Rahmenbedingungen, gesetzlich, Studium, etc.)?

4. Was sollten Krankenhäuser tun, um akademisch ausgebildete Pflegefachpersonen längerfristig zu halten und motiviert zu halten (>5 Jahre)?
   a. Kennen Sie Beispiele guter Praxis?
   b. Was sollte in Deutschland an den Rahmenbedingungen verändert werden, um Krankenhäuser zu unterstützen? (politische Rahmenbedingungen, gesetzlich, Studium, etc.)?

5. Wie sollten hochschulisch qualifizierte Pflegefachpersonen in der Praxis eingesetzt werden?
    a. Gibt es spezielle Tätigkeitsbereiche für diese Pflegefachpersonen? Wenn ja, wie sehen diese aus/können Sie mir Beispiele nennen?
        i. Unterscheidung in Bachelor / Master

6. Gibt es zum Schluss irgendetwas, was Sie noch hinzufügen möchten oder was Sie denken, das wichtig ist zum Thema hochschulisch ausgebildete Pflegepersonen und Einbindung in die klinische Pflegepraxis?

Ende des Interviews: *Ich danke Ihnen für Ihre Teilnahme. Wenn Sie nach diesem Interview noch Fragen haben sollten, oder Ihnen noch etwas einfällt, können Sie sich gerne an mich wenden - die Kontaktinformationen finden Sie auf dem Informationsblatt zur Teilnahme.*

Nach dem Interview: **Kurzfragebogen ausfüllen lassen**

Maier CB, *et al. BMJ Open* 2023; 13:e073879. doi: 10.1136/bmjopen-2023-073879

Technische Universität Berlin

Interview guide 6 (in English) for international experts

For international experts and stakeholders (U.S., Belgium, other countries as relevant)

Introduction: *The number of nurses with a Bachelor´s degree or higher has increased over the recent past in Germany. Yet, the predominant educational background is still a 3-year vocational training. Currently it is estimated that around 2% of all nurses in university hospitals hold a Bachelor´s degree or higher (also referred to as academically educated nurses). Nurses with a Master´s degree and Advanced Practice Nurses are even less frequently working in Germany. Hence, it is still fairly new for hospitals in Germany to employ Bachelor- or higher educated nurses. There are questions of how to attract these nurses and how to retain them.*

We would like to learn from other countries with a longer tradition of academically trained nurses, e.g., in BELGIUM/UNITED STATES. What has worked well, what has not worked well? How are hospitals attracting and retaining their Bachelor/Master level nurses? Are there lessons to be shared and good practices?

1. Can you tell us about the current situation of Bachelor and Master-level educated nurses in your country?
   a. Are academically educated nurses the majority in hospitals/healthcare in your country? How many /what proportion of nurses have at least a bachelor's degree?
   b. Are there strategies/plans or recommendations to increase the proportion of academically educated nurses in your country?
      i. If yes, what is the content?
      ii. What measures have been taken?
      iii. What outcomes?
      iv. What lessons learned/what went well/not so well?

2. Where do academically educated nurses work in your country?
   a. Do most academically educated nurses work in hospitals or ambulatory care? Why?
   b. How many academically educated nurses work outside clinical care? Why?
   c. How many work outside healthcare? Why?

3. We would now like to learn more about academically educated nurses working in hospital settings. Do you know of strategies of good practices of hospitals that are using to attract this workforce to their hospitals?
   a. What exactly was done?
   b. What outcomes?
   c. What lessons learned / what went well / not so well?

4. We would now like to learn more about hospitals' strategies in your country to retain academically educated nurses for a longer period (let's say over 5 years). Do you know of strategies of good practices of hospitals that are using to keep this workforce motivated in hospitals?
   a. What exactly was done?
   b. What outcomes?
   c. What lessons learned / what went well / not so well?

Technische Universität Berlin

5. Are there any barriers that hospitals are commonly experiencing that prevent them to employing or to keeping more academically educated nurses?
    a. What exactly?
    b. Why
    c. What outcomes?
    d. What lessons learned?

6. Are there any facilitating factors/measures or strategies that hospitals used to employing or to keeping more academically educated nurses?
    a. What exactly?
    b. Why
    c. What outcomes?
    d. What lessons learned?

7. Are there country-specific measures that have been taken to increase the number of Bachelor educated nurses WORKING in clinical care?
    a. What was done?
    b. What outcomes?
    c. What lessons learned? what went well / not so well?

8. What else would you like to share that we haven't discussed yet, but is relevant when it comes to increasing and retaining academically educated nurses?
    a. What exactly?
    b. Why?
    c. What lessons learned?
    d. What lessons for Germany?

End of the interview: *Thank you for your participation. If you have any questions after this interview, or if you think of anything else, please feel free to contact me. The contact information is on the participation information sheet.*

After the interview: ask interviewees to fill in short survey
