# Supplementary material for: Recruiting and retaining bachelor qualified nurses in German hospitals (BSN4Hospital): protocol of a mixed-methods design
Source: BMJ Open. 2023 Aug 10;13(8):e073879. doi: 10.1136/bmjopen-2023-073879 (PMC10423778; doi:10.1136/bmjopen-2023-073879)
Supplement: Author's manuscript [file bmjopen-2023-073879.draft_revisions.pdf]

BMJ Open is committed to open peer review. As part of this commitment we make the peer review history of every article we publish publicly available.

When an article is published we post the peer reviewers' comments and the authors' responses online. We also post the versions of the paper that were used during peer review. These are the versions that the peer review comments apply to.

The versions of the paper that follow are the versions that were submitted during the peer review process. They are not the versions of record or the final published versions. They should not be cited or distributed as the published version of this manuscript.

BMJ Open is an open access journal and the full, final, typeset and author-corrected version of record of the manuscript is available on our site with no access controls, subscription charges or pay-per-view fees (http://bmjopen.bmj.com).

If you have any questions on BMJ Open's open peer review process please email
info.bmjopen@bmj.com

BMJ Open

Recruiting and retaining Bachelor qualified nurses in German hospitals (BSN4Hospital): Protocol of a mixed-methods design

Journal:	*BMJ Open*
Manuscript ID	bmjopen-2023-073879
Article Type:	Protocol
Date Submitted by the Author:	20-Mar-2023

Complete List of Authors:	Maier, Claudia B.; Technische Universitat Berlin, Department of Healthcare Management ; University of Pennsylvania, Center for Health Outcomes and Policy Research, School of Nursing<br>Köppen, Julia; Technische Universität Berlin, Department of Healthcare Management<br>Kleine, Joan; Technische Universitat Berlin, Department of Healthcare Management<br>McHugh, Matthew; University of Pennsylvania, School of Nursing, Center for Health Outcomes and Policy Research<br>Sermeus, Walter; KU Leuven, Public Health & Primary Care; KU Leuven<br>Aiken, Linda; University of Pennsylvania, School of Nursing;

Keywords:	Nursing Care, Human resource management < HEALTH SERVICES ADMINISTRATION & MANAGEMENT, Organisation of health services < HEALTH SERVICES ADMINISTRATION & MANAGEMENT

SCHOLARONE™
Manuscripts

Recruiting and retaining Bachelor qualified nurses in German hospitals (BSN4Hospital): Protocol of a mixed-methods design

Authors

Claudia B. Maier[1 2*], Julia Köppen[1], Joan Kleine[1], Matthew D. McHugh[2], Walter Sermeus[3], Linda H. Aiken[2]

[1)] Department of Healthcare Management,
Technische Universität Berlin
Straße des 17. Juni 135, H80
10623 Berlin,
Germany

[2)] Center for Health Outcomes and Policy Research,
University of Pennsylvania
School of Nursing, Claire Fagin Hall
418 Curie Blvd
Philadelphia, PA 19104
United States

[3)] Leuven Institute for Healthcare Policy,
KU Leuven
Kapucijnenvoer 35 blok d - box 7001
3000 Leuven
Belgium

*Corresponding author

E-mails

c.maier@tu-berlin.de
julia.koeppen@tu-berlin.de
joan.kleine@tu-berlin.de
mchughm@nursing.upenn.edu
walter.sermeus@kuleuven.be

For peer review only - http://bmjopen.bmj.com/site/about/guidelines.xhtml

laiken@nursing.upenn.edu

ORCID:

Claudia B. Maier. 0000-0002-7734-2258

Julia Köppen. 0000-0001-7941-641X

Joan Kleine

Matthew D. McHugh. 0000-0002-1263-0697

Walter Sermeus. 0000-0002-5915-1845

Linda H. Aiken

For peer review only

For peer review only - http://bmjopen.bmj.com/site/about/guidelines.xhtml

Abstract

Introduction. Many countries in Europe are facing a shortage of nurses and seek effective recruitment and retention strategies. The nursing workforce is increasingly diverse in its educational background, ranging from diploma to Bachelor and Master educated nurses. This study analyses recruitment and retention strategies for academically educated nurses (minimum Bachelor level) compared with diploma nurses in innovative German hospitals; and lessons on recruitment and retention taking an international perspective.

Methods and analysis. The study will apply a mixed methods design, including qualitative and quantitative methods. The qualitative study will include semi-structured interviews among hospital managers, nurses, students, and stakeholders in Germany as well as expert interviews internationally. The quantitative, cross-sectional study consists of a survey among professional nurses in German hospitals. The aim is to compare the retention, job satisfaction and work engagement between academic nurses (Bachelor / Master) and diploma educated nurses. Inclusion criteria are hospitals with a higher than average proportion of Bachelor nurses or innovative recruitment measures, work environment or retention strategies in place. Analyses will be conducted in several phases, first in parallel, then combined via triangulation: the parallel analysis technique will be analyse the qualitative and quantitative data separately via content analyses (interviews) and descriptive, bivariate and multivariate analyses (survey). Subsequently, data sources will be collectively analyzed via a triangulation matrix focusing on developing thematic exploratory clusters at three systemic levels: micro, meso and macro level. The analyses will be relevant for generating lessons for clinical nursing, management, and policy.

Ethics and dissemination. Ethics approval was obtained by the Charité Ethics Committee. Several dissemination channels will be used, including publications and presentations for the scientific community, nursing management, clinical nurses and the wider public in Germany and internationally.

Registration. n/a

Key words. Nursing care, Human resource management, BSN, organisation of health services, recruitment, retention, hospital

Strengths and limitations

For peer review only - http://bmjopen.bmj.com/site/about/guidelines.xhtml

- Mixed methods study on recruitment strategies and retention in German ‘innovator’ hospitals for academically educated nurses (Bachelor / Master) compared with diploma nurses
- Qualitative study on recruitment and retention with in-depth lessons, covering management, nurses, students, stakeholders and international experts
- Quantitative survey to quantify the scale of intention-to-leave among Bachelor / Master educated nurses compared to diploma nurses (primary endpoint) and work engagement, job satisfaction and recruitment and retention measures (secondary endpoints)
- One strength is the focus on ‘innovative’ hospitals with comparatively high proportion of Bachelor educated nurses, a limitation is that it is not representative of all German hospitals
- The cross-sectional design is a limitation as to the causality of the findings

For peer review only

For peer review only - http://bmjopen.bmj.com/site/about/guidelines.xhtml

1. Introduction

Several countries in Europe and globally are facing a nursing shortage which has been exacerbated by the Covid-19 pandemic (1). Effective recruitment and retention strategies for this workforce are therefore of high relevance in hospitals and other healthcare settings in many countries (2). At the same time, the nursing workforce has become more diverse in its educational background, comprising increasing proportions of Bachelor (BSN), Master (MSN) and in some countries, even PhD educated nurses. This has been a result of nursing educational reforms over the past two decades often triggered by the Bologna process in Europe (3, 4). While some countries have moved the primary nursing education entirely to Bachelor level, others have Bachelor education and vocational education co-existing, e.g., in Germany and several other countries (4).

A wide range of interventions influence the recruitment and retention of nurses and other health professionals (2, 5, 6). A literature review of 23 studies found that a positive, relational leadership style and supportive work environments were associated with nurses' retention to stay (5). A review and multiple case study approach (2) on recruitment and retention for health professionals in Europe suggested that bundles of interventions were more effective than single measures but based on few high-quality evaluations across Europe (ibid.). Successful strategies to attract and retain newly qualified nurses were identified as internship or residency programmes, orientation and transition to practice programmes with a mentor component, whilst the length varied considerably (6). Most reviews either subsumed all health professionals (2) or focused on nurses (5, 6), yet there is limited research on recruitment and retention for Bachelor and higher educated nurses compared with nurses with no academic qualification.

Intention-to-leave (ITL) is a common indicator to measure nurses' willingness to leave the current employment or the nursing workforce. It can also serve as a proxy for retention in hospitals. At the management level, supportive leadership and work environment have been associated with increased retention (5). At the individual nurse level, commitment to the job, resilience, levels of knowledge and confidence, as well as preparation for transition and the expectations regarding supervision were identified as relevant (Collard et al. 2020). In the United States (U.S.), reasons to stay with the current employer were autonomy, payment, and organisational policies, among others (7).

Internationally, few studies have compared ITL among Bachelor (or higher) educated nurses compared with non-academically qualified nurses. A survey in the U.S. found that the higher the educational level (from diploma to doctorate) the lower was the ITL of the nursing profession within the next year (8). In another U.S. study, ITL was 21% of all nurses surveyed in hospitals (9), but the probability was slightly higher for BSN nurses or higher

For peer review only - http://bmjopen.bmj.com/site/about/guidelines.xhtml

qualification. Major differences existed across generations. The probability for ITL was higher for generation Z (born 1997 and later) but lower for Generation X (born 1965-1980) and Boomer (born 1946-1964) in contrast to Millennials (born 1981-1996; reference category) (ibid). Results of a study in South Korea (10) showed that BSN had a higher probability for ITL than nurses with a master's degree or higher. In addition, ITL was higher for younger nurses (26 to 40 years vs. 41 years and older), nurses with less years of work experience, and working in smaller hospitals (<400 vs. ≥800 beds). A study from China among newly graduated nurses showed that the ITL was 6,7%, with a higher proportion of BSN vs. lower qualified nurses (11).

To date, there has been limited research in Germany on academically qualified nurses in hospitals with relation to recruitment and retention. One of the reasons is the small number of nurses with at least Bachelor degree in direct patient care, which was 2.1% in 2018 (12), which is very low compared with many other countries worldwide. In Germany, one cross-sectional study among 273 nurses with at least Bachelor degree found high satisfaction rates but a risk of losing these nurses from direct patient care, as approx. 70% reported being enrolled or planning to enrol in additional studies (13). A position paper (14) suggested that a specific strategy for Bachelor nurses' employment may be a facilitating factor to attract and retain this workforce.

Other countries have shown to have more career pathways and options for academically qualified nurses, e.g. to work as APN (15). In light of the current nursing shortage in Germany (16), identifying strategies to recruit and retain BSN is important for German hospitals. Moreover, previous research has shown that higher proportion of Bachelor and Master educated nurses are associated with improved quality of care, lower mortality, improved job satisfaction and intention to stay (17, 18).

The study BSN4Hospital aims to analyse hospital-level strategies and factors that influence the retention and recruitment of academically qualified nurses (minimum Bachelor) in comparison with nurses with no academic education (diploma) in a selection of 'innovator' hospitals in Germany. The study will quantify ITL as well as job satisfaction and work engagement and influencing factors among Bachelor (and higher educated) nurses compared with diploma educated nurses. Moreover, it will identify international lessons from countries with a longer tradition of academically qualified nurses in clinical care. The overall aim is to identify measures amenable to change to retaining and attracting academically qualified nurses in clinical practice.

For peer review only - http://bmjopen.bmj.com/site/about/guidelines.xhtml

2. Methods and analysis

BSN4Hospital will apply a mixed-methods study, including a qualitative and quantitative research design. The specific aims for the qualitative part of this study are (i) to analyse what hospital-level strategies exist to increase the number of academically qualified nurses in German hospitals, e.g. on recruitment and retention strategies for nurses with BSN and MSN; and (ii) facilitating/hindering factors on implementation. The quantitative part of the study aims (i) to quantify ITL among academically qualified nurses compared with non-academically qualified nurses, as well as their job satisfaction and work engagement; and (ii) to determine which factors are associated with individual as well as hospital-level management factors.

Hospitals will be invited to participate in the study if meeting the following inclusion criteria: (i) a comparatively high percentage of academically qualified nurses (BSN, MSN) defined as over 2.1% identified in earlier research (12) or (ii) having innovative strategies in place to increase or retain the number of academically qualified nurses. Innovative strategies are defined as either single or combined recruitment and retention strategies or broader measures to enhance the work environment. Examples of innovative hospitals to improve the work environment are hospitals participating in the EU-funded Magnet4Europe study (19). The study focuses on the implementation of the U.S. Magnet® model (20) in over 60 hospitals in six European countries, including in Germany (19). Within Germany, 20 German hospitals participate to improve nurses' work environment, including increasing the percentage of academically qualified nurses. In addition, other hospitals with high percentages of academically qualified nurses (e.g. university hospitals) or with innovative recruitment and retention strategies will also qualify to be included.

Patient and public involvement. While there is no public involvement planned, the qualitative design of the study will also cover patient representatives via the German federal patient organisation.

2.1 Qualitative research

The aim of the qualitative research is to identify (i) in a selection of 'innovative' hospitals what strategies are in place for academically educated nurses (management level, e.g. chief nursing officers) (target group 1); (ii) the in-depth views of academically educated nurses (individual level) in what are effective measures to increase the proportion of academically qualified nurses in hospitals (recruitment) and effective retention strategies as well as related barriers and facilitators to implementation (target group 2). In addition, nursing students who

For peer review only - http://bmjopen.bmj.com/site/about/guidelines.xhtml

decided to undertake additional (non-nursing) studies or nurses who left clinical practice will be invited for interviews to understand the drivers that led them to their decision (target group 3). Moreover, experts, key stakeholders and the federal German patient representative organization will be interviewed (target group 4) as well as international experts (target group 5). An in-depth understanding of the different factors at the managerial and individual levels are critical to design effective recruitment and retention strategies.

The interviews will follow semi-structured interview guides which have been developed in five versions tailored to the different target groups. The questions in the interview guides were designed as open-ended questions for each of the identified themes, starting each with an introductory question and several probing questions to obtain additional insights, as necessary. All interview guides are in German, except the interview guide for international experts which was developed in English. In addition, a short survey was developed in German and English to obtain information about the interviewees' background (e.g. title / function and job position, years of work experience, educational background, year of birth). Qualitative data collection will follow the COREC guidelines, which include 32 good practice criteria for interviews and focus groups (21).

2.1.1 Sampling strategy and data collection

The interviews will be conducted in at least six innovative German hospitals following the inclusion criteria. Within the hospitals, managers (Chief Nursing Officers (CNO), nursing directors) as well as BSN, MSN and APN will be recruited. The sampling strategy will use a mix of purposeful sampling to select hospitals with contrasting strategies to maximise variation (e.g. bundle of measures vs. single measures, measures targeted at academic nurses and for sub-groups of BSN, MSN and APN). We aim to conduct at least 30 interviews, but we will extend the number of interviews until data saturation is achieved.

The policy and contextual perspective will be included since experts as well as stakeholders will be invited to the study. Within Germany, experts from policy and nursing management associations will be contacted. In addition, interviews with international experts will be conducted, e.g. CNOs or other experts from Belgium and the U.S., to identify additional, innovative measures and policy lessons. The countries were selected for their co-existence of academically and non-academically educated nurses and a longer tradition of educating and employing BSN. There is also demonstrated positive associations of BSN and MSN/APN with patient outcomes (18, 22).

For peer review only - http://bmjopen.bmj.com/site/about/guidelines.xhtml

The interviews will be conducted in person or digitally, depending on the interviewee's availability. Participants will be informed about the aims and contents of the study, data protection and ethical considerations, and the interview procedure. All participants will be asked to sign a consent form prior to the interview. Recordings will be made with a dictaphone (if in person) or via Zoom (if digital, via end-to-end encryption) provided permission is given. Interviews will be transcribed verbatim by a company with expertise in interview transcriptions. The interviews are expected to last between 45 to 90 minutes.

2.2 Quantitative study

The quantitative part of the study will analyse nurses' ITL as well as job satisfaction, work engagement and additional factors affecting recruitment and retention among nurses, between nurses with and without academic qualifications, as well as for the subgroups of nurses with BSN, MSN and APN. An overview of the contents of the survey and instruments to be used are available in Table 1.

Table 1 Topics and instruments of the BSN4Hospital questionnaire

Topics	Number of questions/ items (+ follow-up)	Validated instrument (Yes/No)	Name of instrument	Source
**Primary outcome measure**				
Intention to leave[a]	1 (+1)	Yes	RN4CAST	[1]
**Secondary outcome measures**				
Job Satisfaction	1	Yes	RN4CAST	[1]
Work engagement	1/ 3	Yes	UWES-3	[2]
**Additional variables**				
Role clarity, job control, performance feedback	1/ 9	Yes	NQPS, QEEW	[3]
Leadership	1/12	Yes	-	[4]
Team involvement	1/ 2	Yes	-	[5]
Last shift	1	Yes	RN4CAST	[6]
Quality and safety	3	Yes	RN4CAST	[6]
Recruitment: Reasons for choosing hospital	1/ 11	No		[7]
Retention: Reasons to stay (minimum 5 years)	1/ 19	No		[7]
Career opportunities in hospital	1/ 7	No		[7]
Expanded roles and tasks	1/ 9	No		[8]
Work attitudes by generation (baby boomer, X, Y, Z)	1/ 4	No		[9]
Sample characteristics (e.g. age, gender, contractual situation)	12 (+1)	No		
Educational background	7 (+3)	No		

Notes: [a] Wording of the question: "If possible, would you leave your current hospital within the next year as a result of job dissatisfaction? If yes, what type of work would you seek?", RN4CAST=Registered Nurse Forcasting. UWES-3: Utrecht Work

For peer review only - http://bmjopen.bmj.com/site/about/guidelines.xhtml

Engagement Score, NQPS=Nordic Questionnaire for Psychosocial Factors at work, QEEW=Questionnaire on the Experience and Evaluation of Work, Source: [1] RN4CAST (23), [2] UWES-3 (24), [3] NQPS, QEEW (25, 26), [4] (27, 28), [5] (27), [6] (23), [7] developed and modified from (2, 13, 29), [8] developed and modified from (30, 31), [9] developed and modified from (32, 33).

The following endpoints will be measured:

Primary outcome measure

- ITL, this item rates the likelihood "to leave the current hospital within the next year as a result of job dissatisfaction" ("yes/no" and if "yes", the follow-up question: "if yes, what type of work would you seek? a) nursing in another hospital, b) nursing, but not in a hospital, or c) non-nursing")

Secondary outcome measures

- job satisfaction, using a single ordinal scale asking "How satisfied are you with your current job?" (1 "very dissatisfied" to 4 "very satisfied")
- work engagement score based on the Utrecht Work Engagement Scale (UWES-3) using a short version of three indicators measuring vigour, dedication and absorption

Additional variables to be included:

- role clarity and job control, leadership, team involvement, staffing on the last shift (self-reported), quality and safety (self-reported), recruitment: reasons to choose a hospital as employer ("ideal hospital") (e.g. reputation, good team work, good work-life-balance, high job security, pay by educational qualification, possibility to opt-out from night-shifts, among others), retention: reasons to stay more than 5 years in a hospital (e.g. work-life-balance, team work, nurse staffing, autonomy in daily work, career possibilities, flexible work hours or possibility for sabbatical, flat hierarchy, supportive leadership, etc), career opportunities, work-life priorities by generations (Boomer, X, Y, Z), sociodemographic information (age, sex), average length of employment in hospital (in months) and information about the educational background of respondents (BSN, MSN, APN, PhD, additional non-academic qualifications).

The survey was piloted internally and externally in 2022: internally three colleagues with expertise in conducting surveys and with a background in nursing or physiotherapy answered the survey and provided in-depth feedback and comments, externally, three nurses working in one hospital in Berlin to which personal contacts existed, provided feedback and

For peer review only - http://bmjopen.bmj.com/site/about/guidelines.xhtml

comments. Moreover, members of the advisory board of the project provided additional feedback on the survey. The feedback was integrated in subsequent rounds.

2.2.1 Sample size

The main endpoint in the quantitative part of the BSN4Hospital study is the ITL (vs. intention to stay) the current hospital. To date, no study in Germany has analysed ITL between academically and non-academically educated nurses using statistical methods and therefore no effect sizes exist for Germany. In the international literature, two studies (8, 10) described small effect sizes for ITL in relation to the qualification level, Cohen's d 0.15 (10) and 0.1 (8). For the calculation of the sample size, therefore we chose an effect size of 0.2 (Cohen's d), which indicates small differences between two groups and is considered relevant. The rationale for this is that recruitment and retention measures should be adapted even for small differences in the ITL between nurses with and without academic degrees. In addition, a test power of 80% wa s chosen, a significance level of 5%, and two-sided hypothesis. The sample size calculation took into account the small number and percentage of academically educated nurses in German hospitals compared to all nurses. In Germany, the proportion of academic nurses in university hospitals was approximately 1.0% in direct patient care and 1.7% overall in 2015 (34), which increased to 2.1% in university hospitals in 2018 in a follow-up survey (12). The proportion of academically educated nurses in general hospitals is not known for Germany, so a conservative value of 1.0% is assumed.

The sampling strategy focuses on nurses working (i) in the 20 German hospitals participating in Magnet4Europe, plus (ii) additional university hospitals with comparatively high proportions of academically educated nurses. The 20 hospitals in the Magnet4Europe study will all be invited to participate (which are 9 university hospitals and 11 general hospitals), as these hospitals are particularly motivated in improving work environments and increasing the proportion of academically educated nurses.

As the basis for the sample size calculation, the data on full-time staff were retrieved from the 2019 quality reports of hospitals (35) for the 20 hospitals participating in Magnet4Europe. The proportion of nursing professionals working part-time was 49% in 2020 (36). On this basis, the number of full-time staff was calculated to number of persons. Following the 2.1% value for university hospitals (12), the mean value for the nine participating university hospitals is 42.3 academic nurses (42.3*9 university hospitals = 380.7 nurses) and in general hospitals 6.6 academic nurses (6.6*11 hospitals = 72.6 nurses; estimated 1% value of academic nurses). This leads to a total of 453.3 academic nurses in 20 hospitals.

For peer review only - http://bmjopen.bmj.com/site/about/guidelines.xhtml

A response rate of 40% to 60% is assumed. With an estimated number of 453 academic nurses in the 20 hospitals, between 181 individuals (40% response rate) and 272 individuals (60% response rate) are expected to participate. Assuming an effect size of 0.2 (with a test power of 80%, a significance level of 5%, and two-sided hypothesis generation), a sample of 250 individuals with academic degrees will be needed. If the response rate is only 40%, additional hospitals will be invited to join the study until at least 250 participants with academic degree (minimum Bachelor level) are reached. In Germany, in addition to the nine university hospitals which participate in the Magnet4Europe study, there are 27 more university hospitals which will be invited for the study. For the second group (non-academic nurses), a sample size of 940 is calculated. If a response rate of 40% to 60% is also assumed for the non-academic nurses, between 2350 (40% response rate) and 1567 (60% response rate) would have to be invited to the survey. These values are considered achievable since a total of 21,700 nursing professionals (full time staff) are employed in the participating hospitals according to the quality reports. The ratio of 250 : 940 (2.5 / 9.4) results from the fact that a very small proportion of nurses in Germany have an academic degree. Although a response rate of 40-60% is considered high, it is assumed that the study is feasible and that the response rate will be achieved. This is based on the following assumptions: first, the 20 hospitals participating in Magnet4Europe are motivated to improve nursing and their work environments in general and focus on recruitment and retention for all nurses and academic nurses. Second, some hospitals are specifically working towards increasing the proportion of academically educated nurses. Third, there is already a well-established contact between the hospitals and the researchers at TU Berlin. Finally, it is assumed that academic nurses are motivated to participate in the online survey, especially since it deals with topics such as ITL vs. intention to stay, work engagement, job satisfaction, among others.

For peer review only

2.2.2 Data collection

The survey will be conducted online (i) in hospitals participating in the Magnet4Europe study and (ii) in additional hospitals, e.g. university hospitals in Germany, with a comparatively higher proportion of academic nurses than the average estimated 1-2%. Data collection is planned for the second quarter of 2023. All professional nurses with a minimum of 3-year qualification (vocational education, BSN, MSN, etc.) are eligible to participate if over 18 years of age. Excluded are nursing assistants and non-nursing professionals. All acute care wards in hospitals will be included, except for nurses working in psychiatry/mental health wards.

For peer review only - http://bmjopen.bmj.com/site/about/guidelines.xhtml

The survey will be conducted via SoSciSurvey (37), an online survey tool for scientific studies. The objectives of the study will be communicated by the project coordinators of the individual hospitals to the health care staff (via posters or flyers), this also includes the URL and further information on how to participate in the survey. Interested nurses can thus participate in the survey directly via the URL. Informed consent to conduct the survey will be obtained from participants prior to the survey; participation without consent is not possible. Each hospital will receive an individual survey link. The survey will be anonymous. In SoSciSurvey, IP addresses are not recorded by default and the option to send reminders is not possible.

2.3 Analysis and triangulation of data

The convergent design is chosen as the theoretical framework construct for this mixed methods study, which aims to collect two different, yet equally relevant data sources that are complementary (38, 39). Thus, it aims to ensure that the strengths of the quantitative and qualitative data are being used in a complementary, and mutually beneficial way (39). In the convergent design, the data are often collected simultaneously or shortly after each other. This will also be done for the BSN4Hospital study.

The analysis and triangulation of the data will follow the parallel analysis approach in mixed methods designs, which first analyses the data separately, and then subsequently via triangulation (38).

2.3.1 Qualitative study: analysis of interviews

The interviews will be analysed following Mayring's qualitative content analysis (40) using a software for qualitative data analysis (e.g. Atlas.ti) in an iterative way. The material will be analysed using the combined, deductive-inductive principle of categorisations and code development. The deductive phase will apply a set of pre-defined major codes to the material (e.g. recruitment, retention, hospital-level strategies, individual factors, policy level, etc.), whereas as part of the inductive phase, new categories will be developed in an iterative process based on the interview material. At least two coders will develop the coding rules and major codes (including inclusions and exclusion criteria) on which basis the major codes and sub-codes will be identified. The process will be documented with a codebook and notes for each interviewee. The analysis of interviews will be conducted according to the consolidated qualitative research reporting criteria (COREC) (21).

For peer review only - http://bmjopen.bmj.com/site/about/guidelines.xhtml

2.3.2 Quantitative study: analysis

Analyses will be performed using a statistical software package, e.g. Stata® or SPSS® involving descriptive, bivariate and multivariate regression to analyse nurses' ITL (primary endpoint) by qualification level (academic vs. non-academic, plus sub-group analyses) and the additional outcome measures job satisfaction and work engagement (secondary endpoints), adjusting for potential confounders (hospital type, bed size, work environment, etc.). The analyses will follow the STROBE guidelines for reporting of cross-sectional, observational studies (41).

2.3.3 Triangulation of the data

The triangulation of the two data sources will follow the "parallel analysis" method, i.e., first conducted separately for the quantitative and qualitative data, which are treated as equally relevant (38). This method of analysis is the most common method described in the literature within mixed methods studies. In a subsequent step, the data are then combined and compared to provide a comprehensive overview of the findings from different perspectives (qualitative: the in-depth insights and experiences of individual nurses, as well as managers and experts and quantitative: large number of academic nurses in "innovative" hospitals;). The process of triangulation will involve an iterative approach, first, comparing the quantitative and qualitative data with each other for similarly vs. contrasting findings and reinforcing or complementary findings. For this purpose, a triangulation matrix will be created (42). The aim is to contextualise the results of the quantitative analysis with findings from the qualitative study; and to understand the qualitative data in the context of the questionnaire survey, to explore in depth issues of implementation and recommendations for practice. This will be done according to thematic exploratory clusters at three systemic levels: micro, meso, and macro level, defined as individual, management, policy- levels. This method of analysis will be particularly relevant for generating recommendations for management and policy-makers.

3. Ethics and dissemination

Ethics approval was obtained from the Charité Ethics Committee (EA2/097/22).

The following dissemination activities are planned: The findings from the interviews and the survey will be published in scientific and non-scientific journals aimed at various target groups, including the scientific community, policy-makers, nursing managers and the nursing profession.

In addition, the results of the study will be presented at national and international conferences. International conferences with an interdisciplinary focus will be targeted as well

For peer review only - http://bmjopen.bmj.com/site/about/guidelines.xhtml

those with a focus on nursing. Dissemination will also involve the management levels of hospitals (CNOs, nursing ward managers, board of directors). To this end, recommendations for nursing management and practice will be developed. These will be made available to participating hospitals and to nursing management associations with a request to disseminate within their networks. In order to address a broader non-scientific public in Germany, short articles or press releases will be written and distributed.

4. Conclusions

This study is expected to be the largest study on academically qualified nurses in German hospitals on innovative recruitment and retention strategies using a mixed methods design. The aim is to inform hospital management, clinical nurses and policy makers on managerial strategies to increase and sustain this workforce and policy implications taking into account national and international policy lessons.

For peer review only

For peer review only - http://bmjopen.bmj.com/site/about/guidelines.xhtml

References

1. Buchan J, Catton H, Shaffer F. Sustain and Retain in 2022 and Beyond. The global nursing workforce and the Covid-19 pandemic.: ICN, CGFNS, ICNM; 2022 January 2022.
2. Kroezen M, Dussault G, Craveiro I, Dieleman M, Jansen C, Buchan J, et al. Recruitment and retention of health professionals across Europe: A literature review and multiple case study research. Health Policy. 2015;119(12):1517-28.
3. Lahtinen P, Leino-Kilpi H, Salminen L. Nursing education in the European higher education area - variations in implementation. Nurse Educ Today. 2014;34(6):1040-47.
4. Praxmarer-Fernandes S, Maier CB, Oikarainen A, Buchan J, Perfilieva G. Levels of education offered in nursing and midwifery education in the WHO European region: multicountry baseline assessment. Public Health Panorama. 2017;3(3):419-30.
5. Cowden T, Cummings G, Profetto-McGrath J. Leadership practices and staff nurses' intent to stay: a systematic review. J Nurs Manag. 2011;19(4):461-77.
6. Brook J, Aitken L, Webb R, MacLaren J, Salmon D. Characteristics of successful interventions to reduce turnover and increase retention of early career nurses: A systematic review. Int J Nurs Stud. 2019;91:47-59.
7. Owens RA, Burwell PM, Petros T. Nurses' and nursing students' intent to stay: The impact of quality of life, work environment, and work satisfaction. Nurs Manage. 2022;53(8):34-43.
8. Nogueras DJ. Occupational commitment, education, and experience as a predictor of intent to leave the nursing profession. Nurs Econ. 2006;24(2):86-93, 55.
9. Koehler T, Olds D. Generational Differences in Nurses' Intention to Leave. West J Nurs Res. 2022;44(5):446-55.
10. Lee MA, Ju YH, Lim SH. A study on the intent to leave and stay among hospital nurses in Korea: A cross-sectional survey. J Nurs Manag. 2020;28(2):332-41.
11. Li Z, Cao J, Wu X, Li F, Zhu C. Intention to leave among newly graduated nurses: A descriptive, multicenter study. J Adv Nurs. 2020;76(12):3429-39.
12. Bergjan M, Tannen A, Mai T, Feuchtinger J, Luboeinski J, Bauer J, et al. Einbindung von Pflegefachpersonen mit Hochschulabschlüssen an deutschen Universitätskliniken: ein Follow-up-Survey. Zeitschrift fur Evidenz, Fortbildung und Qualitat im Gesundheitswesen. 2021;163:47-56.
13. Baumann AL, Kugler C. [Career prospects of graduate Bachelor nursing degree programs - Results of a nationwide study in Germany]. Pflege. 2019;32(1):7-16.
14. German Association of Nurses/DBfK. Position des DBfK zum Einsatz von primärqualifizierten Bachelor of Nursing in der Pflegepraxis. Pressemitteilung [Position of the DBfK on the uptake of primary educated Bachelor of Nursing in clinical nursing. Press release]. 2016.
15. Maier CB, Aiken LH, Busse R. Nurses in advanced roles in primary care: policy levers for implementation. Paris: OECD; 2017.
16. German Federal Agency for Employment. Professional staff shortage analysis: 2021: German Federal Agency for Employment; 2022 [Available from: https://statistik.arbeitsagentur.de/SiteGlobals/Forms/Suche/Einzelheftsuche_Formular.html?nn=27096&topic_f=fachkraefte-engpassanalyse.
17. Aiken LH, Sloane DM, Bruyneel L, Van den Heede K, Griffiths P, Busse R, et al. Nurse staffing and education and hospital mortality in nine European countries: a retrospective observational study. Lancet. 2014;383(9931):1824-30.
18. Aiken LH, Sloane DM, Brom HM, Todd BA, Barnes H, Cimiotti JP, et al. Value of Nurse Practitioner Inpatient Hospital Staffing. Medical Care. 2021;59(10):857-63.
19. Sermeus W, Aiken LH, Ball J, Bridges J, Bruyneel L, Busse R, et al. A workplace organisational intervention to improve hospital nurses' and physicians' mental health: study protocol for the Magnet4Europe wait list cluster randomised controlled trial. BMJ open. 2022;12(7):e059159.
20. American Nurse Credentialing Center (ANCC). ANCC Magnet Recognition Program®: ANA; 2022 [Available from: https://www.nursingworld.org/organizational-programs/magnet/.

For peer review only - http://bmjopen.bmj.com/site/about/guidelines.xhtml

21. Tong A, Sainsbury P, Craig J. Consolidated criteria for reporting qualitative research (COREQ): a 32-item checklist for interviews and focus groups. Int J Qual Health Care. 2007;19(6):349-57.
22. Aiken LH, Sloane D, Griffiths P, Rafferty AM, Bruyneel L, McHugh M, et al. Nursing skill mix in European hospitals: cross-sectional study of the association with mortality, patient ratings, and quality of care. BMJ Quality & Safety. 2016.
23. Sermeus W, Aiken LH, Van den Heede K, Rafferty AM, Griffiths P, Moreno-Casbas MT, et al. Nurse forecasting in Europe (RN4CAST): Rationale, design and methodology. BMC nursing. 2011;10:6.
24. Schaufeli WB, Shimazu A, Hakanen J, Salanova M, De Witte H. An ultra-short measure for work engagement: The UWES-3 validation across five countries. European Journal of Psychological Assessment. 2019;35:577-91.
25. Elo A-L, Dallner M, Gamberale F, Hottinen V, Knardahl S, Lindström K, et al. Validation of the Nordic Questionnaire for Psychological and Social Factors at Work—QPSNordic. Innovative theories, tools, and practices in work and organizational psychology. Ashland, OH, US: Hogrefe & Huber Publishers; 2000. p. 47-57.
26. Veldhoven Mv, Jonge Jd, Broersen S, Kompier M, Meijman T. Specific relationships between psychosocial job conditions and job-related stress: A three-level analytic approach. Work & Stress. 2002;16(3):207-28.
27. Schaufeli WB. Engaging leadership in the job demands-resources model. Career Development International. 2015;20(5):446-63.
28. Rahmadani VG, Schaufeli WB, Stouten J, Zhang Z, Zulkarnain Z. Engaging Leadership and Its Implication for Work Engagement and Job Outcomes at the Individual and Team Level: A Multi-Level Longitudinal Study. Int J Environ Res Public Health. 2020;17(3).
29. Isfort M, Gessenich H, Tucman D. Entry into the profession and retention in nursing in North Rhein Westfalia. An analysis of recruitment, binding and retention factors in the professional field of nursing including the determination of relevant salary structures and data: German Institute for Applied Nursing Research; 2022. Available from: https://www.dip.de/fileadmin/data/pdf/Aktuelles/Berufseinm%C3%BCndung_Studienbericht_.pdf.
30. Bond C, Bruhn H, de Bont A, van Exel J, Busse R, Sutton M, et al. The iMpact on practice, oUtcomes and costs of New roles for health pROfeSsionals: a study protocol for MUNROS. BMJ open. 2016;6(4):e010511.
31. Maier CB, Koppen J, Busse R. Task shifting between physicians and nurses in acute care hospitals: cross-sectional study in nine countries. Hum Resour Health. 2018;16(1):24.
32. Christensen SS, Wilson BL, Edelman LS. Can I relate? A review and guide for nurse managers in leading generations. J Nurs Manag. 2018;26(6):689-95.
33. Abraham O, Baden S. [Mix of generations in nursing - challenges for managers]. Generationenmix in der Pflege — Herausforderung für Führungskräfte. Pflegezeitschrift. 2018;71(1):10-3.
34. Tannen A, Feuchtinger J, Strohbücker B, Kocks A. Survey zur Einbindung von Pflegefachpersonen mit Hochschulabschlüssen an deutschen Universitätskliniken. Stand 2015. State of development of the role of academic nursing staff at German university hospitals in 2015. Zeitschrift fur Evidenz, Fortbildung und Qualitat im Gesundheitswesen. 2017;120(Supplement C):39-46.
35. Federal Joint Committee Germany (GBA). GBA Qualitätsberichte 20192019. Available from: https://www.g-ba-qualitaetsberichte.de/#/search.
36. Federal Statistical Office Germany. Grunddaten der Krankenhäuser [Basic hospital data] 2022;Series 12(6.1.1. 2020).
37. Leiner DJ. SoSci Survey (Version 3.1.06) 2019 [Available from: https://www.soscisurvey.de.
38. Östlund U, Kidd L, Wengström Y, Rowa-Dewar N. Combining qualitative and quantitative research within mixed method research designs: A methodological review. International Journal of Nursing Studies. 2011;48(3):369-83.
39. Tariq S, Woodman J. Using mixed methods in health research. JRSM short reports. 2013;4(6):2042533313479197.

For peer review only - http://bmjopen.bmj.com/site/about/guidelines.xhtml

40. Mayring P. Using qualitative content analysis. In: Flick U, von Kardoff E, I S, editors. A companion to qualitative research. Hamburg: Rowohlt; 2000.
41. von Elm E, Altman DG, Egger M, Pocock SJ, Gøtzsche PC, Vandenbroucke JP. The Strengthening the Reporting of Observational Studies in Epidemiology (STROBE) statement: guidelines for reporting observational studies. J Clin Epidemiol. 2008;61(4):344-9.
42. O'Cathain A, Murphy E, Nicholl J. Three techniques for integrating data in mixed methods studies. BMJ. 2010;341:c4587.

For peer review only

For peer review only - http://bmjopen.bmj.com/site/about/guidelines.xhtml

Authors' contributions:

CBM planned the study and methods and wrote the first draft and subsequent versions of the manuscript. JK and JKL contributed to the planning of the study, wrote and revised sections of the manuscript. MMH, WS and LHH provided guidance for the study, revised sections and provided overall advice for the manuscript. All authors were responsible for approving the final manuscript.

Funding statement: This research received funding by the German Federal Ministry of Education and Research, grant number 01GY2004.

Competing interests statement. The authors declare that they have no competing interests with regard to this study.

Word Count: 3,845 words (without the table and abstract). Should the word count exceed this number, please state this in the cover letter upon submission.

For peer review only

BMJ Open

Recruiting and retaining Bachelor qualified nurses in German hospitals (BSN4Hospital): Protocol of a mixed-methods design

Journal:	*BMJ Open*
Manuscript ID	bmjopen-2023-073879.R1
Article Type:	Protocol
Date Submitted by the Author:	07-Jun-2023
Complete List of Authors:	Maier, Claudia B.; Technische Universitat Berlin, Department of Healthcare Management ; University of Pennsylvania, Center for Health Outcomes and Policy Research, School of Nursing<br>Köppen, Julia; Technische Universität Berlin, Department of Healthcare Management<br>Kleine, Joan; Technische Universitat Berlin, Department of Healthcare Management<br>McHugh, Matthew; University of Pennsylvania, School of Nursing, Center for Health Outcomes and Policy Research<br>Sermeus, Walter; KU Leuven, Public Health & Primary Care; KU Leuven<br>Aiken, Linda; University of Pennsylvania, School of Nursing;
**Primary Subject Heading**:	Nursing
Secondary Subject Heading:	Nursing, Health services research, Health policy
Keywords:	Nursing Care, Human resource management < HEALTH SERVICES ADMINISTRATION & MANAGEMENT, Organisation of health services < HEALTH SERVICES ADMINISTRATION & MANAGEMENT, Hospitals, Health Education

SCHOLARONE™
Manuscripts

Recruiting and retaining Bachelor qualified nurses in German hospitals (BSN4Hospital): Protocol of a mixed-methods design

Authors

Claudia B. Maier[1 2*], Julia Köppen[1], Joan Kleine[1], Matthew D. McHugh[2], Walter Sermeus[3], Linda H. Aiken[2]

[1)] Department of Healthcare Management,
Technische Universität Berlin
Straße des 17. Juni 135, H80
10623 Berlin,
Germany

[2)] Center for Health Outcomes and Policy Research,
University of Pennsylvania
School of Nursing, Claire Fagin Hall
418 Curie Blvd
Philadelphia, PA 19104
United States

[3)] Leuven Institute for Healthcare Policy,
KU Leuven
Kapucijnenvoer 35 blok d - box 7001
3000 Leuven
Belgium

*Corresponding author

E-mails

c.maier@tu-berlin.de
julia.koeppen@tu-berlin.de
joan.kleine@tu-berlin.de
mchughm@nursing.upenn.edu
walter.sermeus@kuleuven.be

For peer review only - http://bmjopen.bmj.com/site/about/guidelines.xhtml

laiken@nursing.upenn.edu

ORCID:

Claudia B. Maier. 0000-0002-7734-2258

Julia Köppen. 0000-0001-7941-641X

Joan Kleine

Matthew D. McHugh. 0000-0002-1263-0697

Walter Sermeus. 0000-0002-5915-1845

Linda H. Aiken

For peer review only - http://bmjopen.bmj.com/site/about/guidelines.xhtml

Abstract

Introduction. Many countries in Europe are facing a shortage of nurses and seek effective recruitment and retention strategies. The nursing workforce is increasingly diverse in its educational background, ranging from 3-year vocational training (diploma) to Bachelor and Master educated nurses. This study analyses recruitment and retention strategies for academically educated nurses (minimum Bachelor), including intention-to-leave, job satisfaction and work engagement compared with diploma nurses in innovative German hospitals; it explores recruitment and retention challenges and opportunities, and identifieslessons on recruitment and retention taking an international perspective.

Methods and analysis. The study will apply a convergent mixed-methods design, including qualitative and quantitative methods. The qualitative study will include semi-structured interviews among hospital managers, nurses, students, and stakeholders in Germany. In addition, expert interviews will be conducted internationally in countries with a higher proportion of Bachelor/Master nurses in hospitals. The quantitative, cross-sectional study will consist of a survey among professional nurses (Bachelor/Master, diploma nurses) in German hospitals. Study settings are hospitals with a higher-than-average proportion of Bachelor nurses or relevant recruitment, work environment or retention strategies in place. Analyses will be conducted in several phases, first in parallel, then combined via triangulation: the parallel analysis technique will analyse the qualitative and quantitative data separately via content analyses (interviews) and descriptive, bivariate and multivariate analyses (survey). Subsequently, data sources will be collectively analysed via a triangulation matrix focusing on developing thematic exploratory clusters at three systemic levels: micro, meso and macro level. The analyses will be relevant for generating lessons for clinical nursing, management, and policy in Germany and internationally.

Ethics and dissemination. Ethics approval was obtained by the Charité Ethics Committee. Several dissemination channels will be used, including publications and presentations for the scientific community, nursing management, clinical nurses and the wider public in Germany and internationally.

Registration. n/a

Key words. Nursing care, Human resource management, Bachelor, Graduate Nursing Education (MeSH), organisation of health services, recruitment, retention, hospital

Strengths and limitations

For peer review only - http://bmjopen.bmj.com/site/about/guidelines.xhtml

- Mixed methods study in selected German hospitals on recruitment and retention for academically educated nurses (Bachelor / Master) compared with diploma nurses
- Qualitative study with in-depth insights, covering managers, nurses, students, stakeholders and international experts
- Quantitative survey to quantify the intention-to-leave among Bachelor / Master educated nurses compared with diploma nurses (primary endpoint) and work engagement, job satisfaction (secondary endpoints)
- One strength is the focus on innovative hospitals with a comparatively high proportion of Bachelor educated nurses or relevant recruitment and retention strategies, a limitation is that it is not representative of all German hospitals
- The cross-sectional design is a limitation as to the causality of the findings

For peer review only

For peer review only - http://bmjopen.bmj.com/site/about/guidelines.xhtml

1. Introduction

Several countries in Europe and globally are facing a nursing shortage which has been exacerbated by the Covid-19 pandemic (1). Effective recruitment and retention strategies for this workforce are therefore of high relevance in hospitals and other healthcare settings in many countries (2). At the same time, the nursing workforce has become more diverse in its educational background, comprising increasing proportions of Bachelor (BSN), Master (MSN) and in some countries, even nurses with a doctorate degree. This has been a result of nursing educational reforms over the past two decades often triggered by the Bologna process in Europe (3, 4). While some countries have moved the primary nursing education entirely to Bachelor level, others have Bachelor education and vocational education co-existing, e.g., in Germany and several other countries (4).

A wide range of interventions influence the recruitment and retention of nurses and other health professionals (2, 5, 6). A literature review of 23 studies found that a positive, relational leadership style and supportive work environments were associated with nurses' intention to stay in their current position (5). A review and multiple case study approach (2) on recruitment and retention for health professionals in Europe suggested that bundles of interventions were more effective than single measures but based on few high-quality evaluations across Europe (ibid.). Successful strategies to attract and retain newly qualified nurses were identified as internship or residency programmes, orientation and transition to practice programmes with a mentor component, whilst the length varied considerably (6). Most reviews either subsumed all health professionals (2) or focused on nurses (5, 6), yet there is limited research on recruitment and retention for Bachelor and higher educated nurses compared with diploma nurses with a 3-year vocational training.

Intention-to-leave (ITL) is a common indicator to measure nurses' willingness to leave the current employment or the nursing workforce. It can also serve as a proxy for retention in hospitals. At the management level, supportive leadership and work environment have been associated with increased retention (5). At the individual nurse level, commitment to the job, resilience, levels of knowledge and confidence, as well as preparation for transition and the expectations regarding supervision were identified as relevant. In the United States (U.S.), reasons to stay with the current employer were autonomy, payment, and organisational policies, among others (7). A theoretical model by Cowden & Cummings identified four key areas associated with Intention-to-Stay, namely management (e.g. leadership, recognition, shared decision-making), organisation-specific characteristics (e.g. career development, staffing), work characteristics (e.g. autonomy, work group cohesion) and individual nurse characteristics (e.g. education, age) (8).

For peer review only - http://bmjopen.bmj.com/site/about/guidelines.xhtml

Internationally, few studies have compared ITL among Bachelor (or higher) educated nurses compared with non-academically educated nurses. A survey in the U.S. found that the higher the educational level (from diploma to doctorate) the lower was the ITL of the nursing profession within the next year (9). In another U.S. study, ITL was 21% of all nurses surveyed in hospitals (10), but the probability was slightly higher for BSN or higher qualification. Major differences existed across generations. The probability for ITL was higher for generation Z (born 1997 and later) but lower for Generation X (born 1965-1980) and Boomer (born 1946-1964) in contrast to Millennials (born 1981-1996; reference category) (ibid). Results of a study in South Korea (11) showed that BSN had a higher probability for ITL than nurses with a master's degree or higher. In addition, ITL was higher for younger nurses (26 to 40 years vs. 41 years and older), nurses with less years of work experience, and nurses working in smaller hospitals (<400 vs. ≥800 beds). A study from China among newly graduated nurses showed that the ITL was 6,7%, with a higher proportion of BSN vs. diploma nurses (12).

To date, there has been limited research in Germany on academically educated nurses in hospitals and their recruitment and retention. One of the reasons is the small number of nurses with at least Bachelor degree in direct patient care, which was estimated to be less than 1% in all settings (13) and 2% in 2018 in university hospitals (14), which is very low compared with many other countries worldwide.

In Germany, one cross-sectional study among 273 nurses with at least Bachelor degree found high satisfaction rates but a risk of losing these nurses from direct patient care, as approx. 70% reported being enrolled or planning to enroll in additional studies (15). A position paper (16) suggested that a specific strategy for Bachelor nurses' employment may be a facilitating factor to attract and retain this workforce.

In Germany, there are two major educational pathways to become a professional nurse: either a 3-year vocational training in nursing (diploma) or Bachelor in nursing (BSN). The 3-year training has been the predominant educational pathway to become a professional nurse in Germany and is usually offered by nursing schools linked to hospitals. Bachelor education takes 3,5 to 4 years at universities of applied science or universities. The successful completion of either the 3-year training or the Bachelor graduation including a state exam are the requirements to work as a professional nurse, also recognised by the European Union (17). BSN are considered professional nurses and officially have no additional roles compared with those of a professional nurse with a 3-year training, however, some hospitals use individual approaches to employ and integrate BSN in practice. Subsequent studies at Master's level (MSN) can be pursued in the field of clinical practice with various sub-

For peer review only - http://bmjopen.bmj.com/site/about/guidelines.xhtml

specialisations (including in advanced practice nursing, community health nursing), in nursing management, education or nursing science.

Other countries have shown to have more career pathways and options for BSN or MSN in clinical care (18). In light of the current nursing shortage in Germany (19), identifying strategies to recruit and retain BSN/MSN is important for German hospitals. Moreover, previous research has shown that higher proportion of BSN/MSN are associated with improved quality of care, lower mortality, improved job satisfaction and intention to stay (20-22).

The study BSN4Hospital aims to analyse strategies and factors that influence the retention and recruitment of BSN/MSN in comparison with diploma nurses in a selection of hospitals in Germany, including ITL, job satisfaction and work engagement. It also covers the in-depth perspectives of managers, staff, students and stakeholders in Germany. Moreover, the study will have an international component, it will explore country- and policy contexts and will identify international lessons from countries with a longer tradition of BSN/MSN in clinical care. The overall aim is to identify measures amenable to change to retaining and attracting academically qualified nurses in clinical practice.

2. Methods and analysis

BSN4Hospital (funded by the German Federal Ministry of Education and Research, 2021-2024) will apply a convergent mixed-methods study, including a qualitative and quantitative research design. The study follows the conceptual approach by Cowden & Cummings, namely management characteristics, organization-specific, work and individual nurse characteristics (8).

Study settings are hospitals with (i) a higher-than-average percentage of nurses with at least BSN education which was estimated to be less than 1% in all hospitals/healthcare settings (13) or hospitals with comparatively high number of BSN as per the 2020 nursing budgets submitted to the German Institute for the remuneration in hospitals (23) and/or (ii) having relevant strategies in place to increase or retain nurses with BSN/MSN. Relevant strategies are defined as either single or combined recruitment and retention strategies or broader measures to enhance the work environment. Examples of hospitals to improve the work environment are hospitals participating in the EU-funded Magnet4Europe study (24). The study focuses on the implementation of the U.S. Magnet® model (25) in over 60 hospitals in six European countries, including in Germany (24). Within Germany, 20 German hospitals

For peer review only - http://bmjopen.bmj.com/site/about/guidelines.xhtml

participate to improve nurses' work environment, including increasing the percentage of academically educated nurses.

2.1 Patient and public involvement.

While there is no direct patient or public involvement planned, the qualitative design of the study will cover patient representation. Most patients are probably not familiar with BSN/MSN due to the relatively low numbers on hospital wards. Yet, patient representatives are expected to be more familiar with topics on recruitment and retention and academisation of nursing and therefore, will be recruited via the German federal patient organisation.

2.2 Qualitative research

The qualitative design of this study will include different target groups to allow for the various perspectives involved in recruitment/retention: hospital managers to cover both leadership and management perspectives, BSN/MSN to cover those directly involved in patient care; students as the next generation of nurses; and stakeholders in Germany and internationally on contextual and policy factors.

The following target groups will be covered: (i) management perspective: chief nursing officers (CNO), nurse managers and other key resource persons to focus on what managerial strategies are in place, lessons for implementation and uptake in practice (ii) BSN/MSN as well as nursing students' working in patient care on effective measures, barriers and facilitators in clinical care. Moreover, experts, key stakeholders from nursing associations or organisations and the federal German patient representative organisation will be interviewed on the main facilitating and hindering factors at policy level. Finally, interviews will also be conducted with international experts (e.g. hospital CNOs from Belgium, the U.S. and other countries) to generate lessons internationally and for Germany. The inclusion of international experts in the qualitative design will generate insights into recruitment and retention challenges and lessons in different healthcare systems. The aim is to learn from countries more advanced than Germany with employing BSN and MSN. We chose Belgium and the U.S. (and may extent to other countries) because these countries having a longer history of BSN and more nursing research (than Germany), but the countries still have the co-existence of BSN and diploma nurses/nurses with vocational training in hospitals which is similar to Germany.

The interviews will follow semi-structured interview guides which have been developed and tailored to the different target groups. The questions in the interview guides were designed as open-ended questions for each of the identified target groups and themes, starting each

For peer review only - http://bmjopen.bmj.com/site/about/guidelines.xhtml

with an introductory question and several probing questions to obtain additional insights, as necessary. All interview guides are in German, except the interview guide for international experts which was developed in English (see Supplement 1). The interview guide for international experts differs in several aspects from the other interview guides. It refers to other countries' experiences, lessons and good practices. Questions will be asked about what strategies and measures have been taken in these countries, with which results and lessons for implementation. The perspectives by international experts can provide valuable comparative insights which may go beyond what would be generated within Germany alone and may show potential transferable strategies of benefit for German hospitals or policy; as well as for other countries with a co-existence of BSN/MSN and diploma nurses.

In addition to the interview guides, a short survey was developed in German and English to obtain information about the interviewees' background (e.g. title / function and job position, years of work experience, educational background, year of birth). Qualitative data collection will follow the COREQ guidelines, which include 32 good practice criteria for interviews and focus groups (26).

2.2.1 Sampling strategy and data collection

The interviews will be conducted in at least six German hospitals following the inclusion criteria. The sampling strategy will use a mix of purposeful sampling to select hospitals with contrasting strategies to maximise variation (e.g. bundle of measures vs. single measures, measures targeted at academically educated nurses and for sub-groups of BSN/MSN). The sampling of national and international experts will also be based on purposeful sampling, combined with network strategies, which is based on the researchers' national and international networks as well as hospital networks. We aim to conduct at least 30-40 interviews, but we will extend the number of interviews until data saturation is achieved. The minimum number of interviewees by target group is as follows: a minimum of 6 interviews with hospital CNOs or other managers (management perspective), minimum of 12 interviews with BSN/MSN working in direct patient care, including nursing students (nurses' perspective), as well as 10-12 interviews with German as well as international stakeholders and experts.

The interviews will be conducted by researchers of the project team who have been trained in conducting interviews and have prior experience in conducting qualitative research. Interviews will take place in person or digitally, depending on the interviewee's availability.

For peer review only - http://bmjopen.bmj.com/site/about/guidelines.xhtml

Participants will be informed about the aims and contents of the study, data protection and ethical considerations, and the interview procedure. All participants will be asked to sign a consent form prior to the interview. Recordings will be made with a dictaphone (if in person) or via Zoom (if digital, via end-to-end encryption) provided permission is given. Interviews will be transcribed verbatim by a company with expertise in interview transcriptions. The interviews are expected to last between 45 to 90 minutes.

2.3 Quantitative study

An overview of the key variables and instruments of the survey are available in Table 1.

For peer review only

Table 1 Topics and instruments of the BSN4Hospital questionnaire

**Topics**	**Number of questions/ items (+ follow-up)**	**Validated instrument (Yes/No)**	**Name of instrument**	**Source**
Primary outcome measure				
Intention to leave[a]	1 (+1)	Yes	RN4CAST	[1]
Secondary outcome measures				
Job Satisfaction	1	Yes	RN4CAST	[1]
Work engagement	1/ 3	Yes	UWES-3	[2]
Additional variables				
Role clarity, job control, performance feedback	1/ 9	Yes	NQPS, QEEW	[3]
Leadership	1/12	Yes	-	[4]
Team involvement	1/ 2	Yes	-	[5]
Last shift	1	Yes	RN4CAST	[6]
Quality and safety	3	Yes	RN4CAST	[6]
Recruitment: Reasons for choosing hospital	1/ 11	No		[7]
Retention: Reasons to stay (minimum 5 years)	1/ 19	No		[7]
Career opportunities in hospital	1/ 7	No		[7]
Expanded roles and tasks	1/ 9	No		[8]
Work attitudes by generation (baby boomer, X, Y, Z)	1/ 4	No		[9]
Sample characteristics (e.g. age, gender, contractual situation)	12 (+1)	No		
Educational background	7 (+3)	No		

Notes: [a] Wording of the question: “If possible, would you leave your current hospital within the next year as a result of job dissatisfaction? If yes, what type of work would you seek?”, RN4CAST=Registered Nurse Forcasting. UWES-3: Utrecht Work Engagement Score, NQPS=Nordic Questionnaire for Psychosocial Factors at work, QEEW=Questionnaire on the Experience and Evaluation of Work, Source: [1] RN4CAST (27), [2] UWES-3 (28), [3] NQPS, QEEW (29, 30), [4] (31, 32), [5] (31), [6] (27), [7] developed and modified from (2, 15, 33), [8] developed and modified from (34, 35), [9] developed and modified from (36, 37).

The following endpoints will be measured:

Primary outcome measure

For peer review only - http://bmjopen.bmj.com/site/about/guidelines.xhtml

- Intention-To-Leave (ITL), this item rates the likelihood “to leave the current hospital within the next year as a result of job dissatisfaction” (“yes/no” and if “yes”, the follow-up question: “if yes, what type of work would you seek? a) nursing in another hospital, b) nursing, but not in a hospital, or c) non-nursing”)

Secondary outcome measures

- job satisfaction, using a single ordinal scale asking, “How satisfied are you with your current job?” (1 “very dissatisfied” to 4 “very satisfied”)
- work engagement score based on the Utrecht Work Engagement Scale (UWES-3) using a short version of three indicators measuring vigour, dedication and absorption

Additional variables to be included:

role clarity and job control, leadership, team involvement, staffing (self-reported), quality and safety (self-reported), recruitment: reasons to choose a hospital as employer (“ideal hospital”) (e.g. reputation, good team work, good work-life-balance, high job security, pay by educational qualification, possibility to opt-out from night-shifts, among others), retention: reasons to stay more than 5 years in a hospital (e.g. work-life-balance, team work, nurse staffing, autonomy in daily work, career possibilities, flexible work hours or possibility for sabbatical, flat hierarchy, supportive leadership, etc), career opportunities, work-life priorities by generations (Boomer, X, Y, Z), sociodemographic information (age, sex), average length of employment in hospital (in years) and information about the educational background of respondents (BSN, MSN, doctorate, additional non-academic specialisations).

The survey was piloted internally and externally in 2022: internally three colleagues with expertise in conducting surveys and with a background in nursing or physiotherapy answered the survey and provided in-depth feedback and comments. Externally, three nurses working in one hospital in Berlin to which personal contacts existed, provided feedback and comments. Moreover, members of the advisory board of the project provided additional feedback on the survey. The feedback was integrated in subsequent rounds.

2.3.1 Sample size

The main endpoint in the quantitative part of the BSN4Hospital study is the ITL (vs. intention to stay) the current hospital. To date, no study in Germany has analysed ITL between BSN/MSN and diploma nurses using statistical methods and therefore no effect sizes exist for Germany. In the international literature, two studies (9, 11) described small effect sizes for ITL in relation to the qualification level, Cohen’s d 0.15 (11) and 0.1 (9). For the calculation of

For peer review only - http://bmjopen.bmj.com/site/about/guidelines.xhtml

the sample size, therefore we chose an effect size of 0.2 (Cohen's d), which indicates small differences between two groups and is considered relevant. The rationale for this is that recruitment and retention measures should be adapted even for small differences in the ITL between nurses with and without academic degrees. In addition, a test power of 80% was chosen, a significance level of 5%, and two-sided hypothesis. The sample size calculation took into account the small number and percentage of BSN/MSN in German hospitals and was estimated at 2% for university hospitals (14) and 1% for all other hospitals (13).

As the basis for the sample size calculation, the data on full-time staff were retrieved from the 2019 quality reports of hospitals (38) for the 20 hospitals participating in Magnet4Europe. The proportion of nursing professionals working part-time was 49% in 2020 (39). On this basis, the number of full-time staff was calculated to number of persons. Following the 2.1% value for university hospitals (14), the mean value for the nine participating university hospitals is 42.3 BSN/MSN (42.3*9 university hospitals = 380.7 nurses) and in general hospitals 6.6 BSN/MSN (6.6*11 hospitals = 72.6 nurses; estimated 1% value of BSN/MSN). This leads to a total of 453.BSN/MSN in 20 hospitals.

A response rate of 40% to 60% is assumed. With an estimated number of 453 BSN/MSN in the 20 hospitals, between 181 individuals (40% response rate) and 272 individuals (60% response rate) are expected to participate. Assuming an effect size of 0.2 (with a test power of 80%, a significance level of 5%, and two-sided hypothesis generation), a sample of 250 individuals with academic degrees will be needed. If the response rate is only 40%, additional hospitals will be invited to join the study until at least 250 participants with academic degree (minimum Bachelor level) are reached. For the second group (diploma nurses), a sample size of 940 is calculated. If a response rate of 40% to 60% is also assumed for the diploma nurses, between 2350 (40% response rate) and 1567 (60% response rate) would have to be invited to the survey. These values are considered achievable since a total of 21,700 nursing professionals (full-time staff) are employed in the participating hospitals according to the quality reports. The ratio of 250: 940 (2.5 / 9.4) results from the fact that a very small proportion of nurses in Germany have an academic degree. Although a response rate of 40-60% is considered high, it is assumed that the study is feasible and that the response rate will be achieved. This is based on the following assumptions: first, the 20 hospitals participating in Magnet4Europe are motivated to improve nursing and their work environments in general and focus on recruitment and retention for all nurses and BSN/MSN. Second, some hospitals are specifically working towards increasing the proportion of BSN/MSN. Third, there is already a well-established contact between the hospitals and the researchers at TU Berlin. Finally, it is assumed that BSN/MSN are

For peer review only - http://bmjopen.bmj.com/site/about/guidelines.xhtml

motivated to participate in the online survey, especially since it deals with topics such as ITL vs. intention to stay, work engagement, job satisfaction, among others.

2.3.2 Data collection

The survey will be conducted online (i) in hospitals participating in the Magnet4Europe study and (ii) in additional hospitals with a comparatively higher proportion of BSN/MSN than the average estimated 1-2%. Data collection is planned for the second and third quarter of 2023. Hospitals will be asked to identify all wards with at least one BSN/MSN, subsequently, all professional nurses with a minimum of 3-year qualification (vocational education, BSN, MSN, etc.) are eligible to participate. Excluded are nursing assistants and non-nursing professionals. Excluded are also psychiatry/mental health wards, because psychiatric care differs substantially from somatic care and was therefore not within the scope of the study.

The survey will be conducted via SoSciSurvey (40), an online survey tool for scientific studies. The objectives of the study will be communicated by the project coordinators of the individual hospitals to the nurses (via posters or flyers), this also includes the URL and further information on how to participate in the survey. Interested nurses can thus participate in the survey directly via the URL. Informed consent to conduct the survey will be obtained from participants prior to the survey; participation without consent is not possible. Each hospital will receive an individual survey link. The survey will be anonymous. In SoSciSurvey, IP addresses are not recorded by default and the option to send reminders is not possible.

2.4 Analysis and triangulation of data

The convergent design is chosen as the theoretical framework construct for this mixed methods study, which aims to collect two different, yet equally relevant data sources that are complementary (41, 42). Thus, it aims to ensure that the strengths of the quantitative and qualitative data are being used in a complementary, and mutually beneficial way (42). In the convergent design, the data are often collected simultaneously or shortly after each other. This will also be done for the BSN4Hospital study.

The analysis and triangulation of the data will follow the parallel analysis approach in mixed methods designs, which first analyses the data separately, and then subsequently via triangulation (41).

2.4.1 Qualitative study: analysis of interviews

For peer review only - http://bmjopen.bmj.com/site/about/guidelines.xhtml

The interviews will be analysed following Mayring's qualitative content analysis (43) using a software for qualitative data analysis (e.g. Atlas.ti) in an iterative way. The material will be analysed using the combined, deductive-inductive principle of categorisations and code development. The deductive phase will apply a set of pre-defined major codes to the material (e.g. recruitment, retention, hospital-level strategies, individual factors, policy level, etc.), whereas as part of the inductive phase, new categories will be developed in an iterative process based on the interview material. At least two coders will develop the coding rules and major codes (including inclusions and exclusion criteria) on which basis the major codes and sub-codes will be identified. The process will be documented with a codebook and notes for each interviewee.

2.4.2 Quantitative study: analysis

Analyses will be performed using a statistical software package, e.g. Stata® or SPSS® involving descriptive, bivariate and multivariate regression to analyse nurses' ITL (primary endpoint) by qualification level (academic vs. non-academic, plus sub-group analyses) and the additional outcome measures job satisfaction and work engagement (secondary endpoints), adjusting for potential confounders (hospital type, bed size, work environment, etc.). The analyses will follow the STROBE guidelines for reporting of cross-sectional, observational studies (44).

2.4.3 Triangulation of the data

The triangulation of the two data sources will follow the "parallel analysis" method, i.e., first conducted separately for the quantitative and qualitative data (41). This method of analysis is the most common method described in the literature within mixed methods studies. In a subsequent step, the data are then combined and compared to provide a comprehensive overview of the findings from different perspectives (qualitative: the in-depth insights and experiences of individual nurses, as well as managers and experts and quantitative: large number of academically educated nurses in selected hospitals). The process of triangulation will involve an iterative approach, first, comparing the quantitative and qualitative data with each other for similarly vs. contrasting findings and reinforcing or complementary findings. For this purpose, a triangulation matrix will be created (45). The aim is to contextualise the results of the quantitative analysis with findings from the qualitative study; and to understand the qualitative data in the context of the questionnaire survey, to explore in depth issues of implementation and recommendations for practice. This will be done according to thematic exploratory clusters at three systemic levels: micro, meso, and macro level, defined as individual, management, policy- levels. This method of analysis will be particularly relevant for generating recommendations for management and policy-makers.

For peer review only - http://bmjopen.bmj.com/site/about/guidelines.xhtml

3. Ethics and dissemination

Ethics approval was obtained from the Charité Ethics Committee (EA2/097/22). Participation is anonymous and voluntary and comes with no benefits for the respondents or interviewees. Neither the hospital nor the researchers will know who took part. The interviews will be pseudonymised so that no conclusions about individuals can be drawn.
The following dissemination activities are planned: The findings from the interviews and the survey will be published in scientific and non-scientific journals aimed at various target groups, including the scientific community, policy-makers, nursing managers and the nursing profession.

In addition, the results of the study will be presented at national and international conferences. International conferences with an interdisciplinary focus will be targeted as well those with a focus on nursing. Dissemination will also involve the management levels of hospitals (CNOs, nursing ward managers, board of directors). To this end, recommendations for nursing management and practice will be developed. These will be made available to participating hospitals and to nursing management associations with a request to disseminate within their networks. In order to address a broader non-scientific public in Germany, short articles or press releases will be written and distributed.

In conclusion, this study is expected to be one of the largest studies on academically qualified nurses in German hospitals on recruitment and retention using a convergent mixed methods design. The study seeks to inform hospital management, clinical nurses and policy makers on strategies to increase and sustain this workforce. In addition, it will identify international lessons and policy implications on recruitment and retention of nurses by educational qualification in hospitals.

For peer review only

For peer review only - http://bmjopen.bmj.com/site/about/guidelines.xhtml

References

1. Buchan J, Catton H, Shaffer F. Sustain and Retain in 2022 and Beyond. The global nursing workforce and the Covid-19 pandemic.: ICN, CGFNS, ICNM; 2022 January 2022.
2. Kroezen M, Dussault G, Craveiro I, Dieleman M, Jansen C, Buchan J, et al. Recruitment and retention of health professionals across Europe: A literature review and multiple case study research. Health Policy. 2015;119(12):1517-28.
3. Lahtinen P, Leino-Kilpi H, Salminen L. Nursing education in the European higher education area - variations in implementation. Nurse Educ Today. 2014;34(6):1040-47.
4. Praxmarer-Fernandes S, Maier CB, Oikarainen A, Buchan J, Perfilieva G. Levels of education offered in nursing and midwifery education in the WHO European region: multicountry baseline assessment. Public Health Panorama. 2017;3(3):419-30.
5. Cowden T, Cummings G, Profetto-McGrath J. Leadership practices and staff nurses' intent to stay: a systematic review. J Nurs Manag. 2011;19(4):461-77.
6. Brook J, Aitken L, Webb R, MacLaren J, Salmon D. Characteristics of successful interventions to reduce turnover and increase retention of early career nurses: A systematic review. Int J Nurs Stud. 2019;91:47-59.
7. Owens RA, Burwell PM, Petros T. Nurses' and nursing students' intent to stay: The impact of quality of life, work environment, and work satisfaction. Nurs Manage. 2022;53(8):34-43.
8. Cowden TL, Cummings GG. Nursing theory and concept development: a theoretical model of clinical nurses' intentions to stay in their current positions. J Adv Nurs. 2012;68(7):1646-57.
9. Nogueras DJ. Occupational commitment, education, and experience as a predictor of intent to leave the nursing profession. Nurs Econ. 2006;24(2):86-93, 55.
10. Koehler T, Olds D. Generational Differences in Nurses' Intention to Leave. West J Nurs Res. 2022;44(5):446-55.
11. Lee MA, Ju YH, Lim SH. A study on the intent to leave and stay among hospital nurses in Korea: A cross-sectional survey. J Nurs Manag. 2020;28(2):332-41.
12. Li Z, Cao J, Wu X, Li F, Zhu C. Intention to leave among newly graduated nurses: A descriptive, multicenter study. J Adv Nurs. 2020;76(12):3429-39.
13. Science Council. HQGplus study on academic qualifications for the healthcare system. Update. Quantitative and qualitative study of the situation in academic programs, teaching, research and care provision2022 [cited 2023 May]. Available from: https://www.wissenschaftsrat.de/download/2022/9541-22.html.
14. Bergjan M, Tannen A, Mai T, Feuchtinger J, Luboeinski J, Bauer J, et al. Einbindung von Pflegefachpersonen mit Hochschulabschlüssen an deutschen Universitätskliniken: ein Follow-up-Survey. Zeitschrift fur Evidenz, Fortbildung und Qualitat im Gesundheitswesen. 2021;163:47-56.
15. Baumann AL, Kugler C. [Career prospects of graduate Bachelor nursing degree programs - Results of a nationwide study in Germany]. Pflege. 2019;32(1):7-16.
16. German Association of Nurses/DBfK. Position des DBfK zum Einsatz von primärqualifizierten Bachelor of Nursing in der Pflegepraxis. Pressemitteilung [Position of the DBfK on the uptake of primary educated Bachelor of Nursing in clinical nursing. Press release]. 2016.
17. European Union. Directive 2013/55/EU of the European Parliament and of the Council amending Directive 2005/36/EC on the recognition of professional qualifications and Regulation (EU) No 1024/2012 on administrative cooperation through the Internal Market Information System ('the IMI Regulation'). Text with EEA relevance. Official Journal of the European Union. 2013;L 354/132.
18. Maier CB, Aiken LH, Busse R. Nurses in advanced roles in primary care: policy levers for implementation. Paris: OECD; 2017.
19. German Federal Agency for Employment. Professional staff shortage analysis: 2021: German Federal Agency for Employment; 2022 [Available from: https://statistik.arbeitsagentur.de/SiteGlobals/Forms/Suche/Einzelheftsuche_Formular.html?nn=27096&topic_f=fachkraefte-engpassanalyse.

For peer review only - http://bmjopen.bmj.com/site/about/guidelines.xhtml

20. Aiken LH, Sloane DM, Bruyneel L, Van den Heede K, Griffiths P, Busse R, et al. Nurse staffing and education and hospital mortality in nine European countries: a retrospective observational study. Lancet. 2014;383(9931):1824-30.
21. Aiken LH, Sloane DM, Brom HM, Todd BA, Barnes H, Cimiotti JP, et al. Value of Nurse Practitioner Inpatient Hospital Staffing. Medical Care. 2021;59(10):857-63.
22. Aiken LH, Sloane D, Griffiths P, Rafferty AM, Bruyneel L, McHugh M, et al. Nursing skill mix in European hospitals: cross-sectional study of the association with mortality, patient ratings, and quality of care. BMJ Quality & Safety. 2016.
23. Institute for the remuneration of hospitals. Nursing Budget 2020. Publication of data according to §6a, 3, 6 of the hospital remuneration law: Institute for the remuneration of hospitals; 2022 [Available from: https://www.g-drg.de/pflegebudget-2020/veroeffentlichung-der-angaben-gemaess-6a-absatz-3-satz-6-khentgg.
24. Sermeus W, Aiken LH, Ball J, Bridges J, Bruyneel L, Busse R, et al. A workplace organisational intervention to improve hospital nurses' and physicians' mental health: study protocol for the Magnet4Europe wait list cluster randomised controlled trial. BMJ open. 2022;12(7):e059159.
25. American Nurse Credentialing Center (ANCC). ANCC Magnet Recognition Program®: ANA; 2022 [Available from: https://www.nursingworld.org/organizational-programs/magnet/.
26. Tong A, Sainsbury P, Craig J. Consolidated criteria for reporting qualitative research (COREQ): a 32-item checklist for interviews and focus groups. Int J Qual Health Care. 2007;19(6):349-57.
27. Sermeus W, Aiken LH, Van den Heede K, Rafferty AM, Griffiths P, Moreno-Casbas MT, et al. Nurse forecasting in Europe (RN4CAST): Rationale, design and methodology. BMC nursing. 2011;10:6.
28. Schaufeli WB, Shimazu A, Hakanen J, Salanova M, De Witte H. An ultra-short measure for work engagement: The UWES-3 validation across five countries. European Journal of Psychological Assessment. 2019;35:577-91.
29. Elo A-L, Dallner M, Gamberale F, Hottinen V, Knardahl S, Lindström K, et al. Validation of the Nordic Questionnaire for Psychological and Social Factors at Work—QPSNordic. Innovative theories, tools, and practices in work and organizational psychology. Ashland, OH, US: Hogrefe & Huber Publishers; 2000. p. 47-57.
30. Veldhoven Mv, Jonge Jd, Broersen S, Kompier M, Meijman T. Specific relationships between psychosocial job conditions and job-related stress: A three-level analytic approach. Work & Stress. 2002;16(3):207-28.
31. Schaufeli WB. Engaging leadership in the job demands-resources model. Career Development International. 2015;20(5):446-63.
32. Rahmadani VG, Schaufeli WB, Stouten J, Zhang Z, Zulkarnain Z. Engaging Leadership and Its Implication for Work Engagement and Job Outcomes at the Individual and Team Level: A Multi-Level Longitudinal Study. Int J Environ Res Public Health. 2020;17(3).
33. Isfort M, Gessenich H, Tucman D. Entry into the profession and retention in nursing in North Rhein Westfalia. An analysis of recruitment, binding and retention factors in the professional field of nursing including the determination of relevant salary structures and data: German Institute for Applied Nursing Research; 2022. Available from: https://www.dip.de/fileadmin/data/pdf/Aktuelles/Berufseinm%C3%BCndung_Studienbericht_.pdf.
34. Bond C, Bruhn H, de Bont A, van Exel J, Busse R, Sutton M, et al. The iMpact on practice, oUtcomes and costs of New roles for health pROfeSsionals: a study protocol for MUNROS. BMJ open. 2016;6(4):e010511.
35. Maier CB, Koppen J, Busse R. Task shifting between physicians and nurses in acute care hospitals: cross-sectional study in nine countries. Hum Resour Health. 2018;16(1):24.
36. Christensen SS, Wilson BL, Edelman LS. Can I relate? A review and guide for nurse managers in leading generations. J Nurs Manag. 2018;26(6):689-95.
37. Abraham O, Baden S. [Mix of generations in nursing - challenges for managers]. Generationenmix in der Pflege — Herausforderung für Führungskräfte. Pflegezeitschrift. 2018;71(1):10-3.

For peer review only - http://bmjopen.bmj.com/site/about/guidelines.xhtml

38. Federal Joint Committee Germany (GBA). GBA Qualitätsberichte 20192019. Available from: https://www.g-ba-qualitaetsberichte.de/#/search.
39. Federal Statistical Office Germany. Grunddaten der Krankenhäuser [Basic hospital data] 2022;Series 12(6.1.1. 2020).
40. Leiner DJ. SoSci Survey (Version 3.1.06) 2019 [Available from: https://www.soscisurvey.de.
41. Östlund U, Kidd L, Wengström Y, Rowa-Dewar N. Combining qualitative and quantitative research within mixed method research designs: A methodological review. International Journal of Nursing Studies. 2011;48(3):369-83.
42. Tariq S, Woodman J. Using mixed methods in health research. JRSM short reports. 2013;4(6):2042533313479197.
43. Mayring P. Using qualitative content analysis. In: Flick U, von Kardoff E, I S, editors. A companion to qualitative research. Hamburg: Rowohlt; 2000.
44. von Elm E, Altman DG, Egger M, Pocock SJ, Gøtzsche PC, Vandenbroucke JP. The Strengthening the Reporting of Observational Studies in Epidemiology (STROBE) statement: guidelines for reporting observational studies. J Clin Epidemiol. 2008;61(4):344-9.
45. O'Cathain A, Murphy E, Nicholl J. Three techniques for integrating data in mixed methods studies. BMJ. 2010;341:c4587.

For peer review only

For peer review only - http://bmjopen.bmj.com/site/about/guidelines.xhtml

Authors' contributions:

CBM planned the study and methods and wrote the first draft and subsequent versions of the manuscript. JK and JKL contributed to the planning of the study, wrote and revised sections of the manuscript. MMH, WS and LHH provided guidance for the study, revised sections and provided overall advice for the manuscript. All authors were responsible for approving the final manuscript.

Funding statement: This research received funding by the German Federal Ministry of Education and Research, grant number 01GY2004.

Competing interests statement. The authors declare that they have no competing interests with regard to this study.

Word Count: 4087 words (without the table and abstract). Should the word count exceed this number, please state this in the cover letter upon submission.

For peer review only

Technische Universität Berlin

Supplement 1. Interview guides: BSN4Hospital Study

Interview guide 1 (hospital managers, e.g. CNOs) in German

Für CNOs/Pflegedirektion in deutschen Krankenhäusern

Einstieg: *Der Anteil der Pflegepersonen mit mindestens Bachelor-Abschluss (auch hochschulisch qualifizierte Pflegefachpersonen genannt), ist in deutschen Krankenhäusern noch gering, im Vergleich zu anderen europäischen Ländern. Ich möchte darum gerne mehr über die Einbindung hochschulisch ausgebildeter Pflegender in Ihrem Krankenhaus erfahren. Vorab möchte ich noch einmal darauf hinweisen, dass ich mit „hochschulisch bzw. akademisch ausgebildet", Pflegepersonen meine, die <u>mindestens</u> einen Bachelor-Abschluss haben, oder auch einen höheren Abschluss, also einen Masterabschluss, wie z.B. Advanced Practice Nurses (APN).*

1. Gibt es in Ihrem Haus eine Strategie oder einen Aktionsplan, um den Anteil des hochschulisch ausgebildete (akademisierten) Pflegefachpersonals zu erhöhen? Wenn ja, was sind die Inhalte?
   a) Wurden für Ihr Haus Ziele formuliert den Anteil hochschulisch ausgebildeter Pflegefachpersonen zu erhöhen? Wenn ja, kennen Sie dieses Ziel/ den Zielwert? (Prozent/ Bis wann?)
   b) Wie setzen Sie dieses Ziel um?
   c) Wie wird der Erfolg gemessen/ Wird ein Monitoring durchgeführt?
   d) Wissen Sie, wie viele akademisch ausgebildete Pflegefachpersonen in Ihrem Haus arbeiten? Hat sich der Anteil in den letzten Jahren verändert?

2. Gibt es in Ihrem Haus Maßnahmen <u>zur Rekrutierung und Einbindung von</u> akademisierten Pflegefachpersonen?
   a) Wie wurden die Maßnahmen ausgewählt und umgesetzt?
   b) Was sehen Sie als förderlich für die Rekrutierung/Gewinnung?
   c) Welche Hindernisse gibt es Ihrer Meinung nach?
   d) Was sollte verbessert werden?

3. Gibt es in Ihrem Haus Maßnahmen zur <u>längerfristigen Bindung</u> von akademisierten Pflegefachpersonen?
   a) Wie wurden die Maßnahmen ausgewählt und umgesetzt?
   b) Wie gut gelingt es aus Ihrer Sicht Ihrer Klinik hochschulisch ausgebildete Pflegefachpersonen längerfristig zu halten?
   c) Was sehen Sie als förderlich für die langfristige Bindung?
   d) Welche Hindernisse gibt es Ihrer Meinung nach?
   e) Was sollte verbessert werden?

4. Wie werden hochschulisch qualifizierte Pflegefachpersonen in Ihrem Haus in der Praxis eingesetzt?
   a) Gibt es spezielle Tätigkeitsbereiche für diese Pflegefachpersonen? Wenn ja, wie sehen diese aus/können Sie mir Beispiele nennen?

For peer review only

Technische Universität Berlin

b) Gibt es Aufgaben, die ausschließlich von akademisierten Pflegefachpersonen in Ihrem Haus übernommen werden? Welche sind das?

c) Gibt es ein Mentoring-Programm/spezifisches Einarbeitungsprogramm für akademisiertes Pflegepersonal?

5. Was sind Ihrer Meinung nach die Hauptfaktoren/-bedingungen, warum sich akademisierte Pflegefachpersonen für die Arbeit in Ihrem Krankenhaus entscheiden?

a) Was bietet das Krankenhaus dieser Berufsgruppe?

b) Gibt es Angebote in Ihrem Haus, die besonders anziehend auf hochschulisch ausgebildete Pflegende wirken?

c) Was müsste noch verbessert werden?

Wir haben uns bisher über Ihr Haus unterhalten. Jetzt erweitern wir den Blick auf die Situation in Deutschland.

6. Was denken Sie, wie steht Ihr Haus im Vergleich zu anderen Krankenhäusern in Deutschland da (in Bezug auf die Rekrutierung und langfristige Bindung hochschulisch ausgebildeter Pflegefachpersonen)?
    a. Was läuft gut?
    b. Was läuft weniger gut?
    c. Was könnte insgesamt an deutschen Krankenhäusern verbessert werden, um hochschulisch ausgebildete Pflegefachpersonen zu rekrutieren und zu halten?
    d. Gibt es hinderliche Faktoren oder Rahmenbedingungen in Deutschland, die es den Krankenhäusern erschweren, akademisches Pflegefachpersonal zu gewinnen und zu halten?
    e. Gibt es förderliche Faktoren in Deutschland, die es den Krankenhäusern erleichtern, akademisches Pflegefachpersonal zu gewinnen und zu halten?

7. Was können deutsche Krankenhäuser aus den bisherigen Erfahrungen mit der Rekrutierung und Binding von akademisiertem Pflegepersonal für die Zukunft lernen?
    a. Was sollten deutsche Krankenhäuser generell bedenken, wenn Sie akademisches Pflegepersonal binden möchten?

8. Gibt es zum Schluss irgendetwas, was Sie noch hinzufügen möchten oder was Sie denken, das wichtig ist, für das Thema Gewinnung und Bindung hochschulisch ausgebildeter Pflegepersonen?

Ende des Interviews: *Ich danke Ihnen für Ihre Teilnahme. Wenn Sie nach diesem Interview noch Fragen haben sollten, oder Ihnen noch etwas einfällt, können Sie sich gerne an mich wenden - die Kontaktinformationen finden Sie auf dem Informationsblatt zur Teilnahme.*

Nach dem Interview: **Kurzfragebogen ausfüllen lassen**

For peer review only - http://bmjopen.bmj.com/site/about/guidelines.xhtml

Technische Universität Berlin

Interview guide 2 (in German) for BSN/MSN

Für Pflegepersonen mit BSN/ MSN in deutschen Krankenhäusern

Einstieg: *Der Anteil der Pflegepersonen mit mindestens Bachelor-Abschluss (auch hochschulisch qualifizierte Pflegefachpersonen genannt), ist in deutschen Krankenhäusern noch gering, im Vergleich zu anderen europäischen Ländern. Mich interessieren darum Ihre Erfahrungen und Motivation als akademisch ausgebildete*r Pflegende*r. Vorab möchte ich noch einmal darauf hinweisen, dass ich mit „hochschulisch bzw. akademisch ausgebildet", Pflegepersonen meine, die <u>mindestens</u> einen Bachelor-Abschluss haben, oder auch einen höheren Abschluss, also einen Masterabschluss wie z.B. Advanced Practice Nurses (APN).*

1. Als erstes würde mich interessieren, wie lange Sie schon in Ihrem Krankenhaus tätig sind?
   a. Welchen Abschluss haben Sie und seit wann?
   b. Warum haben Sie sich für einen Studienabschluss in der Pflege entschieden?
   c. Haben Sie vor weiter zu studieren/promovieren/habilitieren? Warum?

2. Warum arbeiten Sie in genau diesem Krankenhaus?
   a. Warum haben Sie sich für die Arbeit in diesem Krankenhaus entschieden?
      i. Gab es ausschlaggebende Faktoren, die Sie überzeugt haben?
      ii. Hat Sie etwas besonders angesprochen? Wenn ja, was?
   b. Können Sie mir sagen, was Sie besonders motiviert in Ihrem Krankenhause zu arbeiten und warum?
   c. Gibt es etwas, das Sie frustriert? Wenn ja, was ist das und warum?
   d. Wie lange beabsichtigen Sie in diesem Krankenhaus tätig zu sein?
      iii. Was Sind Ihre beruflichen Pläne?
      iv. Inwieweit wurde Ihre berufliche Zukunft mit der Leitungsebene/Pflegedirektion besprochen?

3. Haben Sie als Pflegeperson mit Bachelor/Master-Abschluss eine spezielle Rolle im Krankenhaus?
   a. Wie unterscheiden sich Ihre Tätigkeiten zu Pflegefachpersonen ohne Studium?
   b. Wie sieht Ihre Zusammenarbeit im Team aus?
   c. Wie zufrieden bzw. unzufrieden sind Sie mit Ihrer Rolle und warum?
   d. Was würden Sie gerne ändern?

4. Welche Maßnahmen sollte es in Ihrer Klinik geben, um mehr Pflegefachpersonen mit Bachelor- oder Masterabschluss zu gewinnen und längerfristig zu halten?
   a. Sollten sich die Maßnahmen zu Gewinnung von akademisiertem Pflegepersonal von den Maßnahmen zur langfristigen Bindung unterscheiden? Können Sie mir Beispiele nennen?
      i. Welche Themen sind wichtig, wenn es um die langfristige Bindung von akademischen Pflegefachpersonen geht?

Wir haben uns bisher über Ihre Erfahrungen in diesem Haus unterhalten. Jetzt erweitern wir den Blick auf die Situation in Deutschland.

5. Was denken Sie, wie steht Ihr Haus im Vergleich zu anderen Krankenhäusern in Deutschland da?

For peer review only - http://bmjopen.bmj.com/site/about/guidelines.xhtml

Technische Universität Berlin

a. Haben Sie bereits in anderen Krankenhäusern gearbeitet?
b. Was war da anders?
c. Was lief gut/schlecht?

6. Was sollte sich in Deutschland ändern, um mehr hochschulisch ausgebildete Pflegefachpersonen für die klinische Pflege zu gewinnen?
   a. Welche Faktoren sind hinderlich/ förderlich?
   b. Was sollte verbessert werden und wie?

7. Abschließend würde ich Sie gerne bitten, sich vorzustellen, dass Sie auf der Suche nach einer neuen Stelle in einem anderen Krankenhaus sind. Was würden Sie sich in Bezug auf die neue Stelle und Tätigkeit wünschen?
   a. Was würde Sie überzeugen in einem Krankenhaus anzufangen?

8. Gibt es zum Schluss irgendetwas, das Sie noch hinzufügen möchten oder was Sie denken, das wichtig ist zum Thema Gewinnung und Bindung hochschulisch ausgebildeter Pflegefachpersonen?

Ende des Interviews: *Ich danke Ihnen für Ihre Teilnahme. Wenn Sie nach diesem Interview noch Fragen haben sollten, oder Ihnen noch etwas einfällt, können Sie sich gerne an mich wenden - die Kontaktinformationen finden Sie auf dem Informationsblatt zur Teilnahme.*

Nach dem Interview: **Kurzfragebogen ausfüllen lassen**

For peer review only

Technische Universität Berlin

Interview guide 3 (in German) for students

Für Studierende in einem Pflegestudiengang mit (erster) praktischer Erfahrung

Einstieg: *Der Anteil der Pflegepersonen mit mindestens Bachelor-Abschluss ist in deutschen Krankenhäusern noch gering, im Vergleich zu anderen europäischen Ländern. Mich interessieren darum Ihre Erfahrungen und Beweggründe,* **warum Sie sich für ein Pflegestudium** *entschieden haben. Vorab möchte ich noch einmal darauf hinweisen, dass ich mit „hochschulisch bzw. akademisch ausgebildet", Pflegepersonen meine, die* <u>*mindestens*</u> *einen Bachelor-Abschluss haben, oder auch einen höheren Abschluss, also einen Masterabschluss wie z.B. Advanced Practice Nurses (APN).*

1. Als erstes würde mich interessieren, wie lange Sie schon studieren?
    a. Welchen Abschluss streben Sie an und wann werden Sie diesen voraussichtlich erreichen?
    b. Warum haben Sie sich für einen Studiengang in der Pflege entschieden?
    c. Haben Sie bereits einen Berufs- oder Studienabschluss?
    d. Arbeiten Sie noch nebenbei oder haben Sie während Ihres Studiums nebenbei gearbeitet? Welchen Nebenjob üben Sie derzeit aus?

2. Hatten Sie schon Praxiseinsätze (wie viele Einsätze, unterschiedliche Organisationen, welche Fachbereiche?) Wie erleben Sie die Arbeit/Tätigkeit als Studierende im Krankenhaus?
    a. Was war/ist gut? Was nicht so gut?
    b. Auf welcher Station haben Sie zuletzt gearbeitet, bzw. wo sind Sie derzeit tätig? *(Fachrichtung/internistisch/chirurgisch/ITS/etc.)*?

3. Warum haben Sie sich für dieses Krankenhaus als Arbeitgeber (Praxispartner) entschieden?
    a. Gab es ausschlaggebende Faktoren, die Sie überzeugt haben?
    b. Hat Sie etwas besonders angesprochen? Wenn ja, was?
    c. Können Sie mir sagen, was Sie besonders motiviert, in Ihrem Krankenhause zu arbeiten und warum?
    d. Gibt es etwas, das Sie frustriert? Wenn ja, was ist das und warum?

4. In welchem Bereich möchten Sie nach Abschluss Ihres Studiums arbeiten?
    a. Wenn nicht klinischer Bereich, dann fragen, ob es für Sie infrage käme, in die klinische Pflege zu bleiben?
        i. Welche Bedingungen müssten dafür erfüllt sein?
        ii. Was müsste sich in deutschen Krankenhäusern ändern?
        iii. Was müsste sich generell in der Pflege ändern?
        iv. Was müsste sich in Deutschland ändern?
    b. Haben Sie vor weiter zu studieren/promovieren/habilitieren? Warum?

5. Haben Pflegefachpersonen mit Bachelor/Master-Abschluss eine spezielle Rolle in Ihrem Krankenhaus?
    a. Wie unterscheiden sich die Tätigkeiten zu Pflegefachpersonen ohne Studium?
    b. Welche Strategien, Maßnahmen oder Konzepte gibt es in Ihrem Krankenhaus, um Pflegefachpersonen mit Bachelor- oder Masterabschluss in der Pflegepraxis zu integrieren?
    c.

For peer review only - http://bmjopen.bmj.com/site/about/guidelines.xhtml

Technische Universität Berlin

6. Welche Maßnahmen sollte es in Ihrer Klinik geben, um mehr Pflegefachpersonen mit Bachelor- oder Masterabschluss zu gewinnen und längerfristig zu halten?
    a. Sollten sich die Maßnahmen zu Gewinnung von hochschulisch ausgebildetem Pflegepersonal von den Maßnahmen zur langfristigen Bindung unterscheiden? Können Sie mir Beispiele nennen?
        I. Welche Themen sind wichtig, wenn es um die langfristige Bindung von akademischen Pflegefachpersonen geht? Probing: wie kann man diese Pflegefachpersonen länger an einer Klinik halten, d.h. mind. länger als 5 Jahre - oder sogar 10 Jahre?

Wir haben uns bisher über Ihre Erfahrungen in diesem Haus unterhalten. Jetzt erweitern wir den Blick auf die Situation in Deutschland.

7. Was denken Sie, wie steht Ihr Haus im Vergleich zu anderen Krankenhäusern in Deutschland da?
    d. Haben Sie bereits in anderen Krankenhäusern gearbeitet?
    e. Was war da anders?
    f. Was lief gut/schlecht?

8. Was sollte sich in Deutschland ändern, um mehr hochschulisch ausgebildete Pflegefachpersonen für die klinische Pflege zu gewinnen?
    c. Welche Faktoren sind hinderlich/ förderlich?
    d. Was sollte verbessert werden und wie?

9. Abschließend würde ich Sie gerne bitten, sich vorzustellen, dass Sie auf der Suche nach einer neuen Stelle in einem anderen Krankenhaus sind. Was würden Sie sich in Bezug auf die neue Stelle und Tätigkeit wünschen?
    b. Was würde Sie überzeugen in einem Krankenhaus anzufangen?

10. Gibt es zum Schluss irgendetwas, dass Sie noch hinzufügen möchten oder was Sie denken, das wichtig ist zum Thema Gewinnung und Bindung hochschulisch ausgebildeter Pflegefachpersonen?

Ende des Interviews: *Ich danke Ihnen für Ihre Teilnahme. Wenn Sie nach diesem Interview noch Fragen haben sollten, oder Ihnen noch etwas einfällt, können Sie sich gerne an mich wenden - die Kontaktinformationen finden Sie auf dem Informationsblatt zur Teilnahme.*

Nach dem Interview: **Kurzfragebogen ausfüllen lassen**

For peer review only - http://bmjopen.bmj.com/site/about/guidelines.xhtml

Technische Universität Berlin

Interview guide 4 (in German) for "leavers"

Pflegefachpersonen, die sich trotz Pflegestudium gegen eine klinische Tätigkeit entschieden haben oder sich gegen ein Pflegestudium (und gegen eine klinische Tätigkeit) entschieden haben.

Einstieg: *Der Anteil der Pflegepersonen, mit mindestens Bachelor-Abschluss, ist in deutschen Krankenhäusern noch gering, im Vergleich zu anderen europäischen Ländern. Mich interessieren darum Ihre Erfahrungen und Beweggründe, [JE nach Interviewteilnehmer*in anpassen] warum Sie für ein Pflegestudium aber gegen die klinische Tätigkeit/ warum Sie sich für ein Studium ohne Pflegebezug und gegen die klinische Tätigkeit entschieden haben. Vorab möchte ich noch einmal darauf hinweisen, dass ich mit „hochschulisch bzw. akademisch ausgebildet", Pflegepersonen meine, die mindesten einen Bachelor-Abschluss haben, oder auch einen höheren Abschluss, also einen Masterabschluss wie z.B. Advanced Practice Nurses (APN).*

1. Sie haben eine Ausbildung in der Pflege und haben in der Vergangenheit als Pflegefachperson in der klinischen Pflege im Krankenhaus gearbeitet. Wie haben Sie die Arbeit/Tätigkeit als Pflegefachperson im Krankenhaus erlebt?
   a. Was war gut? Was nicht so gut?
   b. Warum haben Sie sich damals für diesen Beruf entschieden?
   c. Auf welcher Station haben Sie zuletzt gearbeitet *(Fachrichtung/internistisch/chirurgisch/ITS/etc.)*?
   d. Welche Strategien, Maßnahmen oder Konzepte gab es in den Krankenhäusern, in denen Sie zuvor gearbeitet haben, um Pflegefachpersonen mit Bachelor- oder Masterabschluss in der Pflegepraxis zu integrieren?

2. Sie haben ja auch einen Studienabschluss bzw. studieren derzeit, oder?
   a. Können Sie erläutern, was Ihre Beweggründe waren, ein Studium anzufangen?
   b. Was haben Sie studiert bzw. studieren Sie derzeit?
   c. Warum haben Sie sich für den Studiengang entschieden?

3. Die Anzahl der Studiengänge der Fachrichtung Pflege hat über die letzten Jahre hinweg zugenommen, auch die Anzahl der Absolvent*innen. Sie haben sich *[JE nach Interviewteilnehmer*in anpassen: für ein Pflegestudium bzw. für ein Studium ohne Pflegeschwerpunkt]* entschieden. Mich interessiert, was die Gründe dafür waren.
   a. Warum haben Sie sich für bzw. gegen einen Pflegestudiengang entschieden?
   b. Haben Sie vor weiter in dem von Ihnen gewählten Bereich zu studieren/promovieren/habilitieren? Warum?
   c. Falls Pflege und noch studierend: In welchem Bereich möchten Sie nach Abschluss Ihres Studiums arbeiten?
   d. Welchen Beruf üben Sie derzeit aus?

4. In Deutschland hat der Wissenschaftsrat bereits 2012 empfohlen, dass 10-20% eines Ausbildungsjahrgangs in der Pflege hochschulisch ausgebildet werden sollen. Wie ist Ihre Meinung dazu?

For peer review only - http://bmjopen.bmj.com/site/about/guidelines.xhtml

a. Sollte Ihrer Meinung nach der Anteil hochschulisch ausgebildeter Pflegefachpersonen in der klinischen Pflege in Krankenhäusern erhöht werden? Wenn ja, warum?
b. Was sind Barrieren? Hinderliche Rahmenbedingungen?
c. Was sind förderliche Faktoren?
d. Was sollte sich in Deutschland/deutschen Krankenhäusern verändern? (Arbeitsumfeld; tarifliche Eingruppierung; Arbeitszeiten; Mitspracherecht; etc.)?

5. Welche Maßnahmen sollte es geben, um mehr Pflegefachpersonen mit Bachelor- oder Masterabschluss zu gewinnen und längerfristig zu halten?
   a. Was sollten Krankenhäuser tun, um akademisch ausgebildete Pflegefachpersonen erfolgreich zu rekrutieren?
   b. Welche Faktoren sind hinderlich/ förderlich?
   c. Was sollte sich in Deutschland verändern?
   d. Was sollte verbessert werden und wie?

6. Falls nicht in einem Pflegestudium: Abschließend möchte ich Sie fragen, ob es für Sie infrage käme, in die klinische Pflege zurückzukehren?
   a. Welche Bedingungen müssten dafür erfüllt sein?
   b. Was müsste sich in deutschen Krankenhäusern ändern?
   c. Was müsste sich generell in der Pflege ändern?
   d. Was müsste sich in Deutschland ändern?

7. Gibt es zum Schluss irgendetwas, das Sie noch hinzufügen möchten oder was Sie denken, das wichtig ist zum Thema Gewinnung und Bindung hochschulisch ausgebildeter Pflegefachpersonen?

Ende des Interviews: *Ich danke Ihnen für Ihre Teilnahme. Wenn Sie nach diesem Interview noch Fragen haben sollten, oder Ihnen noch etwas einfällt, können Sie sich gerne an mich wenden - die Kontaktinformationen finden Sie auf dem Informationsblatt zur Teilnahme.*

Nach dem Interview: **Kurzfragebogen ausfüllen lassen**

For peer review only - http://bmjopen.bmj.com/site/about/guidelines.xhtml

Technische Universität Berlin

Interview guide 5 (in German) for stakeholders/experts

Für Expert*innen und Stakeholders in Deutschland (z.B. Interessenvertretung, Patient*innenvertreter)

Einstieg: *Der Anteil hochschulisch ausgebildeter Pflegefachpersonen, also mit mindestens Bachelor-Abschluss, ist in deutschen Krankenhäusern noch gering, v.a. im Vergleich zu anderen europäischen Ländern. Es ist in Deutschland immer noch eine relativ neue Entwicklung: die Pflegestudiengänge, Einbindung in die klinische Praxis, Tätigkeitsfelder der Pflegefachpersonen mit Bachelor- und Masterabschluss, Vergütung, usw. Dies sind alles Themen, die derzeit aktuell sind. Ich möchte darum heute gerne mehr von Ihnen erfahren, welche Voraussetzungen geschaffen werden sollten, damit hochschulisch ausgebildete Pflegefachpersonen gerne und motiviert in der klinischen Pflege arbeiten möchten, wie Arbeitgeber (bspw. Krankenhäuser) attraktiv werden und diese Pflegefachpersonen auch längerfristig halten. Vorab möchte ich noch einmal darauf hinweisen, dass ich mit „hochschulisch bzw. akademisch ausgebildet", Pflegepersonen meine, die mindestens einen Bachelor-Abschluss haben, oder einen höheren Abschluss, also einen Masterabschluss wie z.B. Advanced Practice Nurses (APN).*

1. In Deutschland hat der Wissenschaftsrat bereits 2012 empfohlen, dass 10-20% eines Ausbildungsjahrgangs in der Pflege hochschulisch ausgebildet werden sollen. Dies ist ja nun bereits 10 Jahre her. Was hat sich Ihrer Meinung nach seither getan?
    a. Was lief gut?
    b. Was waren die Herausforderungen? Was lief nicht gut?
    c. Wie könnte verbessert werden?

2. Die Anzahl der Studiengänge Pflege hat ja über die letzten Jahre hinweg zugenommen, auch die Anzahl der Absolvent*innen. Jedoch arbeitet trotz allem nur ein geringer Prozentsatz in der klinischen Pflege (ca. 2-3% aller Pflegefachpersonen in Universitätsklinika). Sollte der Anteil erhöht werden? Wenn ja, warum und wie?
    a. Was sind Barrieren? Hinderliche Rahmenbedingungen?
    b. Gab es förderliche Faktoren?
    c. Was sollte in Deutschland verändert werden? (politische Rahmenbedingungen, gesetzlich, Studium, etc.)?

3. Wir möchten nun den Schwerpunkt auf Krankenhäuser legen: was sollten Krankenhäuser tun, um akademisch ausgebildete Pflegefachpersonen erfolgreich zu rekrutieren?
    a. Kennen Sie Beispiele guter Praxis?
    b. Was sollte in Deutschland an den Rahmenbedingungen verändert werden, um Krankenhäuser zu unterstützen? (politische Rahmenbedingungen, gesetzlich, Studium, etc.)?

4. Was sollten Krankenhäuser tun, um akademisch ausgebildete Pflegefachpersonen längerfristig zu halten und motiviert zu halten (>5 Jahre)?
    a. Kennen Sie Beispiele guter Praxis?
    b. Was sollte in Deutschland an den Rahmenbedingungen verändert werden, um Krankenhäuser zu unterstützen? (politische Rahmenbedingungen, gesetzlich, Studium, etc.)?

Technische Universität Berlin

5. Wie sollten hochschulisch qualifizierte Pflegefachpersonen in der Praxis eingesetzt werden?
   a. Gibt es spezielle Tätigkeitsbereiche für diese Pflegefachpersonen? Wenn ja, wie sehen diese aus/können Sie mir Beispiele nennen?
      i. Unterscheidung in Bachelor / Master

6. Gibt es zum Schluss irgendetwas, was Sie noch hinzufügen möchten oder was Sie denken, das wichtig ist zum Thema hochschulisch ausgebildete Pflegepersonen und Einbindung in die klinische Pflegepraxis?

Ende des Interviews: *Ich danke Ihnen für Ihre Teilnahme. Wenn Sie nach diesem Interview noch Fragen haben sollten, oder Ihnen noch etwas einfällt, können Sie sich gerne an mich wenden - die Kontaktinformationen finden Sie auf dem Informationsblatt zur Teilnahme.*

Nach dem Interview: **Kurzfragebogen ausfüllen lassen**

For peer review only

For peer review only - http://bmjopen.bmj.com/site/about/guidelines.xhtml

Technische Universität Berlin

Interview guide 6 (in English) for international experts

For international experts and stakeholders (U.S., Belgium, other countries as relevant)

Introduction: *The number of nurses with a Bachelor´s degree or higher has increased over the recent past in Germany. Yet, the predominant educational background is still a 3-year vocational training. Currently it is estimated that around 2% of all nurses in university hospitals hold a Bachelor´s degree or higher (also referred to as academically educated nurses). Nurses with a Master´s degree and Advanced Practice Nurses are even less frequently working in Germany. Hence, it is still fairly new for hospitals in Germany to employ Bachelor- or higher educated nurses. There are questions of how to attract these nurses and how to retain them.*

We would like to learn from other countries with a longer tradition of academically trained nurses, e.g., in BELGIUM/UNITED STATES. What has worked well, what has not worked well? How are hospitals attracting and retaining their Bachelor/Master level nurses? Are there lessons to be shared and good practices?

1. Can you tell us about the current situation of Bachelor and Master-level educated nurses in your country?
   a. Are academically educated nurses the majority in hospitals/healthcare in your country? How many /what proportion of nurses have at least a bachelor's degree?
   b. Are there strategies/plans or recommendations to increase the proportion of academically educated nurses in your country?
      i. If yes, what is the content?
      ii. What measures have been taken?
      iii. What outcomes?
      iv. What lessons learned/what went well/not so well?

2. Where do academically educated nurses work in your country?
   a. Do most academically educated nurses work in hospitals or ambulatory care? Why?
   b. How many academically educated nurses work outside clinical care? Why?
   c. How many work outside healthcare? Why?

3. We would now like to learn more about academically educated nurses working in hospital settings. Do you know of strategies of good practices of hospitals that are using to attract this workforce to their hospitals?
   a. What exactly was done?
   b. What outcomes?
   c. What lessons learned / what went well / not so well?

4. We would now like to learn more about hospitals' strategies in your country to retain academically educated nurses for a longer period (let's say over 5 years). Do you know of strategies of good practices of hospitals that are using to keep this workforce motivated in hospitals?
   a. What exactly was done?
   b. What outcomes?
   c. What lessons learned / what went well / not so well?

For peer review only - http://bmjopen.bmj.com/site/about/guidelines.xhtml

Technische Universität Berlin

5. Are there any barriers that hospitals are commonly experiencing that prevent them to employing or to keeping more academically educated nurses?
   a. What exactly?
   b. Why
   c. What outcomes?
   d. What lessons learned?

6. Are there any facilitating factors/measures or strategies that hospitals used to employing or to keeping more academically educated nurses?
   a. What exactly?
   b. Why
   c. What outcomes?
   d. What lessons learned?

7. Are there country-specific measures that have been taken to increase the number of Bachelor educated nurses WORKING in clinical care?
   a. What was done?
   b. What outcomes?
   c. What lessons learned? what went well / not so well?

8. What else would you like to share that we haven't discussed yet, but is relevant when it comes to increasing and retaining academically educated nurses?
   a. What exactly?
   b. Why?
   c. What lessons learned?
   d. What lessons for Germany?

End of the interview: *Thank you for your participation. If you have any questions after this interview, or if you think of anything else, please feel free to contact me. The contact information is on the participation information sheet.*

After the interview: ask interviewees to fill in short survey

For peer review only
